# PRMT1-mediated PGK1 arginine methylation promotes colorectal cancer glycolysis and tumorigenesis

**DOI:** 10.1038/s41419-024-06544-6

**Published:** 2024-02-24

**Authors:** Hao Liu, Xintian Chen, Pengfei Wang, Miaolei Chen, Chuyin Deng, Xingyou Qian, Jin Bai, Zhongwei Li, Xiangyang Yu

**Affiliations:** 1 School of Medicine, Nankai University, Tianjin, China; 2 Cancer Institute, Xuzhou Medical University, Xuzhou, Jiangsu China; 3 Department of Gastroenterology, the Affiliated Hospital of Guangdong Medical University, Zhanjiang, Guangdong China; 4 Department of Gastroenterology, the First People’s Hospital of Shuyang County, Suqian, Jiangsu China; 5 Center of Clinical Oncology, the Affiliated Hospital of Xuzhou Medical University, Xuzhou, Jiangsu China; 6 Jiangsu Center for the Collaboration and Innovation of Cancer Biotherapy, Cancer Institute, Xuzhou Medical University, Xuzhou, Jiangsu China; 7 Laboratory of Tumor Epigenetics, Department of Pathophysiology, School of Basic Medical Sciences, Wannan Medical College, Wuhu, Anhui China; 8 Department of Gastrointestinal Surgery, the Hospital of Integrated Chinese and Western Medicine, Tianjin, China

**Keywords:** Cancer metabolism, Methylation, Phosphorylation, Methylases, Drug development

## Abstract

Many types of cancer cells, including colorectal cancer cells (CRC), can simultaneously enhance glycolysis and repress the mitochondrial tricarboxylic acid (TCA) cycle, which is called the Warburg effect. However, the detailed mechanisms of abnormal activation of the glycolysis pathway in colorectal cancer are largely unknown. In this study, we reveal that the protein arginine methyltransferase 1 (PRMT1) promotes glycolysis, proliferation, and tumorigenesis in CRC cells. Mechanistically, PRMT1-mediated arginine asymmetric dimethylation modification of phosphoglycerate kinase 1 (PGK1, the first ATP-producing enzyme in glycolysis) at R206 (meR206-PGK1) enhances the phosphorylation level of PGK1 at S203 (pS203-PGK1), which inhibits mitochondrial function and promotes glycolysis. We found that PRMT1 and meR206-PGK1 expression were positively correlated with pS203-PGK1 expression in tissues from colorectal cancer patients. Furthermore, we also confirmed that meR206-PGK1 expression is positively correlated with the poor survival of patients with colorectal cancer. Our findings show that PRMT1 and meR206-PGK1 may become promising predictive biomarkers for the prognosis of patients with CRC and that arginine methyltransferase inhibitors have great potential in colorectal cancer treatment.

## Introduction

The incidence and mortality of colorectal cancer (CRC) continue to increase [1]. Early diagnosis and treatment are effective methods to reduce colorectal cancer mortality [2], and fully analyzing the molecular mechanism of CRC occurrence and development has great significance for early diagnosis and treatment.

Enhanced glycolysis significantly promotes tumor progression [3]. Targeting glycolysis is very attractive for tumor therapy. Currently, a variety of drugs targeting glucose transporters, hexokinases, and lactate dehydrogenase in the process of glycolysis have entered clinical trials [4]. Several articles have reported abnormal glycolysis enhancement in CRC [5, 6], but the detailed mechanism of abnormal glycolysis has not yet been clarified, and the targeted glycolysis process is a potential scheme for the treatment of CRC.

Protein methyltransferases play a key role in the progression of tumors, and arginine methyltransferases (PRMTs) and lysine methyltransferases mainly catalyze protein methylation [7, 8]. Arginine methyltransferase inhibitors have been reported to significantly inhibit pancreatic cancer deterioration, and the US FDA approved tazemetostat, the first inhibitor of the lysine methyltransferase EZH2, for the treatment of epithelioid sarcoma and follicular lymphoma [9, 10]. PRMT1 is the most active arginine methyltransferase in the PRMT family, as well as the most dominant type I PRMT in mammalian cells [11]. In recent years, the role of PRMT1 in the metabolism of hepatocellular carcinoma has been widely discussed. PRMT1-mediated phosphoglycerate dehydrogenase methylation can promote serine accumulation in hepatocellular carcinoma [12], and can enhance glycolysis in hepatocellular carcinoma cells and promote the progression of liver cancer by methylating thymidine kinase and remodeling the actin cytoskeleton [13, 14]. We previously reported that PRMT1 catalyzes EZH2 methylation modification and promotes the progression of breast cancer [15–17]. Abnormal glycolysis activation is often found in CRC [18], but it has not been clarified whether PRMT1 is involved in abnormal glycolysis in CRC.

Here, we report that PRMT1 enhances colorectal cancer cell glycolysis and　tumorigenesis. Mechanistically, PRMT1 binds to PGK1 and methylates it at R206 (meR206-PGK1), which leads to increased ERK-mediated phosphorylation of PGK1 at S203, which phosphorylates pyruvate kinase and inhibits PDH complex-dependent pyruvate utilization in mitochondria, leading to increased glycolytic activity and tumor progression. Moreover, we also showed that PRMT1 expression and meR206K-PGK1 expression are positively correlated; meR206-PGK1 expression is also positively correlated with that of pS203-PGK1, and high expression of meR206-PGK1 is positively associated with a poor prognosis in CRC patients. Our research provides a new mechanism underlying the abnormal glycolysis mediated by PRMT1 and shows the potential value of using methyltransferase inhibitors for CRC treatments.

## Materials and Methods

### Patients and sample collection

The colorectal cancer tissue microarrays (TMAs) containing 96 cases of CRC tissue specimens, purchased from Avilabio Company, were used for PRMT1, meR206-PGK1, and pS203-PGK1 staining. TMAs slides included 207 pairs of tissue specimens of CRC tissues and the corresponding adjacent normal colorectal tissues from CRC patient cohorts enrolled at the Affiliated Hospital of Xuzhou Medical University from 2010 to 2015. The patient studies were conducted according to the Declaration of Helsinki, and the use of these specimens and data for research purposes was approved by the Ethics Committee of the Hospital.

### Cell Culture and cell treatment

HCT116, DLD1, HEK293T and LOVO cells, obtained from the cell bank of the Chinese Academy of Sciences, were cultured in DMEM (KGL1207-500, KeyGEN BioTECH) or F12 (KGL1304-500, KeyGEN BioTECH) supplemented with 10% fetal bovine serum, and incubated in a 37 °C humidified incubator with 5% CO_2_.

siLenFect reagent (Thermo Fisher Scientific Inc, USA) was used to deliver small interfering RNAs (siRNAs, 50 nM) into the CRC cells. All siRNAs were purchased from GenePharma Technology (Shanghai, China). The sequences of negative control and siRNAs are shown in Supplementary Materials and Methods. Lipofectamine 2000 (Life Technologies) was used for plasmid transfections according to the manufacturer’s instructions.

### Western blot analysis and antibodies

Western blot was performed as previously described [19]. Specific primary antibodies against PRMT1 (11279-1-AP, Proteintech), PGK1 (17811-1-AP, Proteintech), GAPDH (60004-1-AP, Proteintech), ADMA **(**13522 S, Cell Signaling Technology), Flag (M29998, Abmart), pS203-PGK1 (SAB487P, Signalway Antibody), meR206-PGK1 (SAB487P-1, Signalway Antibody), PDHA1 (A22081, Abclonal); p-PDHA1 (AP1022, Abclonal); PDHK1 (A0834, Abclonal); p-PDHK1 (11596, Signalway Antibody) ERK1/2 (4695 S, Cell Signaling Technology, short for ERK), Phospho- ERK1/2 (Thr202/Tyr204) (4370 S, Cell Signaling Technology, short for p-ERK) were used for western blot assays. ERK specific inhibitor SCH772984 and PRMT1 inhibitor GSK3368715 were purchased from MedChemExpress (HY-50846, HY-128717A, MCE).

The anti-ADMA-R206-PGK1 (anti-meR206-PGK1) antibody was raised against the region near R206 asymmetric dimethylarginine site of PGK1. The asymmetric di-methylated synthetic peptide [C-NYFAKALESPE(R-Me2,asym)PFLA] was used for immunization in the mice. The antibody was generated in Gl biochem company (Shanghai, China).

### Immunohistochemistry (IHC) staining

IHC assays were performed as previously reported [20]. Retrieval buffer (EDTA, pH 9.0) was used for heat-induced epitope retrieval. The dilution ratio of the primary antibody is as follows, PRMT1, meR206-PGK1, and pS203-PGK1 antibodies were used with 1:100 dilution, and PGK1, Ki-67 (12202 S, CST) antibodies were used with 1:200 dilution. IHC assessment methods were described in the Supplementary Materials and Methods.

### Seahorse assays, glucose uptake, and lactate production

Seahorse Bioscience Extracellular Flux Analyzer (XF96, Seahorse Bioscience Inc., North Billerica, MA, USA) was used to monitor glycolysis and mitochondrial function by measuring extracellular acidification rate (ECAR) and oxygen consumption rate (OCR) as described in manufacturer’s instructions. Glucose uptake and lactate production were performed using the glucose assay kit (Shanghai Rongsheng Biotech, Shanghai, China) and lactate assay kit (Shanghai Rongsheng Biotech, Shanghai, China) following the manufacturer’s instructions as previously described [21, 22], the detailed procedure was described in Supplementary Materials and Methods.

### Cell proliferation and colony formation assays

Cell Counting Kit-8 (CCK-8, VC5001L-500T, VICMID) and colony formation assays was used for proliferation analysis [23], shown as in Supplementary Materials and Methods.

### Stable cell line generation

Stable cells were generated using lentivirus [15]. The detailed procedure was described in the Supplementary Materials and Methods.

### Animal work

BALB/c nude mice (6–8 weeks old) were purchased from Beijing Vital River Laboratory Animal Technology Co., Ltd. (Beijing, China). All animal experiments were approved by the Animal Care and Use Committee at Xuzhou Medical University. For the GSK3368715 treatment, HCT116 cells (4×10^6^) were inoculated subcutaneously. When the tumor reached about 50 mm^3^, mice were randomly divided into the control group (PBS), and GSK3368715 treatment group (GSK3368715), with 6 mice in each group. The GSK3368715 treatment group was given GSK3368715 (100 mg/kg) intraperitoneally once a day for two weeks, and the control group was given solvent control. The tumor growth was observed and recorded. The difference in tumor volume and tumor weight between the control group and the treatment group was recorded and analyzed. For Flag-PGK1-WT and Flag-PGK1-R206K subcutaneous tumor model, group Flag-PGK1-WT and Flag-PGK1-R206K HCT116 cells (4×10^6^) were inoculated subcutaneously. The difference in tumor volume and tumor weight were recorded and analyzed.

### Molecular dynamics simulations and structure analysis

The 3D structure from PDB ID: 2WZB was employed [24]. PGK1 protein was solvated in ~175,000 TIP3P water molecules with 150 mM KCl in a 192×192×192 Å box. The system was built using the CHARMM program with the CHARMM36 force field for all molecules [25]. The system was equilibrated for 55 ns using the NAMD2.12 program package under the periodic orthorhombic boundary conditions applied in all directions with the time step of 2 fs [26]. The NPT ensemble was used for both simulations with pressure at 1 atm and temperature at 310.15 K. Long-range electrostatic interactions were treated by the particle mesh Ewald (PME) algorithm and nonbonded interactions were switched off at 10–12 Å [27].

### Statistical analysis

Statistical analyses were conducted using SPSS 20.0 software (SPSS Inc., Chicago, IL, USA) and GraphPad Prism 7. Data were presented as the mean squared error (SEM). *p* < 0.05 was considered statistically significant.

## Results

### PRMT1 enhances colorectal cancer cell glycolysis

**Fig. 1:**
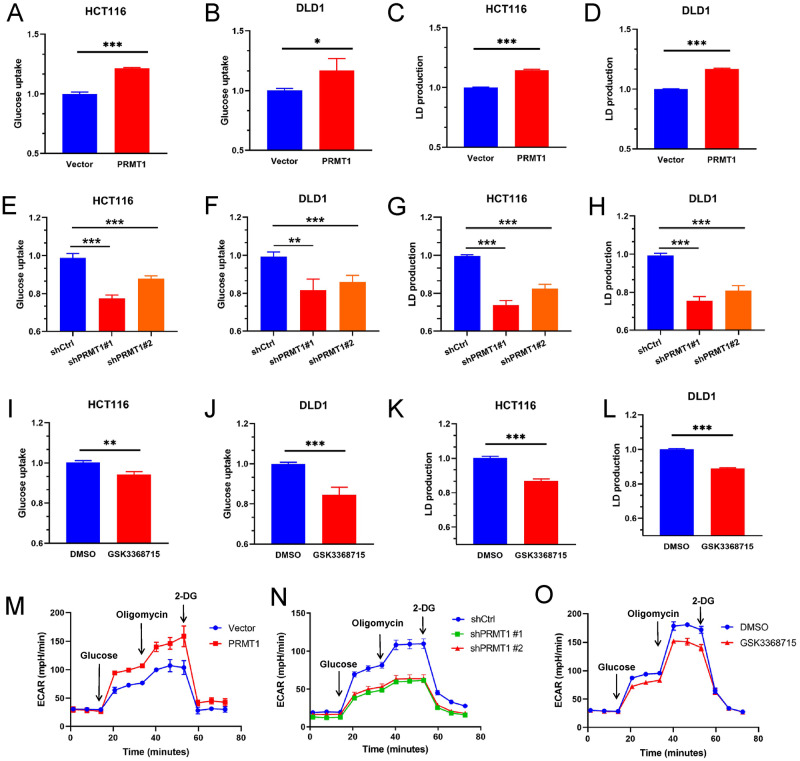
PRMT1 enhances colorectal cancer cell glycolysis. **A**–**L** The glucose uptake kit and lactate production kit were used to analyze the glucose uptake and lactate production when PRMT1 was overexpressed **(A**–**D)** or knocked down **(E**–**H)** in HCT116 cells and DLD1 cells or when HCT116 and DLD1 cells were treated with GSK3368715 (3.1 nM, 48 hours) **(I**–**L)**. **M**–**O** Extracellular acidification rates (ECAR) were analyzed when PRMT1 was stable overexpressed **(M)**, knocked down **(N)** in HCT116 cells or when HCT116 cells were treated with GSK3368715 **(O)** by Seahorse Bioscience Extracellular Flux Analyzer. The working concentration of the drugs showed in **M**–**O** was Glucose (10 μM), Oligomycin (1 μM), and 2-DG (50 μM). GSK3368715 treatment: 3.1 nM for 24 h. Data are represented as mean ± SEM of three independent experiments, and *p < 0.05, **p < 0.01, ***p < 0.001 (Student’s t-test).

To explore whether PRMT1 is involved in the abnormal glycolysis process of colorectal cancer, we overexpressed or knocked down PRMT1 in HCT116 and DLD1 cells to investigate its potential effects on glucose metabolism (Fig. S1). The excessive expression of PRMT1 resulted in increased glucose uptake and lactate production (Fig. 1A–D). PRMT1 deficiency led to decreased glucose uptake and lactate production (Fig. 1E–H), and PRMT1 inhibitor GSK3368715 treatment (a type I protein arginine methyltransferase inhibitor that inhibits asymmetric dimethylation (ADMA) modification) had similar results compared with PRMT1 deficiency (Fig. 1I–L). Consequently, extracellular acidification (ECAR) was increased when PRMT1 was overexpressed. However, the ECAR was decreased after PRMT1 was knocked down or treated with GSK3368715 in HCT116 cells (Fig. 1M–O). Taken together, our data strongly suggest that PRMT1 enhances colorectal cancer cell glycolysis.

### PRMT1 catalyzes PGK1-ADMA at the R206 residue

Changes in the expression of metabolic enzymes are achieved with the occurrence and development of tumors [28], and varieties of metabolic enzymes participate in maintaining the homeostasis of the metabolic process [29]. As an arginine methyltransferase, PRMT1 regulates the expression of target genes by catalyzing arginine methylation modification of target proteins [30]. As our data show, PRMT1 could enhance glycolysis in colorectal cancer cells, and we speculated that there might be glycolytic-related enzymes bound to PRMT1.

**Fig. 2:**
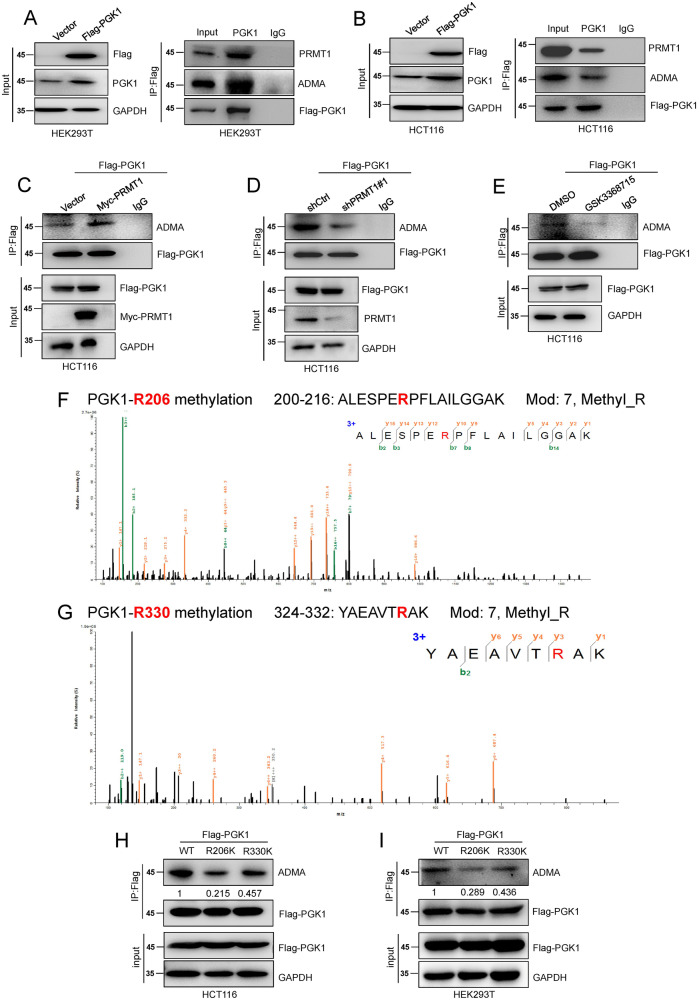
PRMT1 catalyzes PGK1-ADMA at the R206 residue. **A**–**E** HEK293T and HCT116 cells were transfected with exogenous Flag-PGK1 (**A**, **B**). Anti-FLAG beads were used for immunoprecipitation to detect the mutual binding between PRMT1 and PGK1, and the methylation changes of ADMA were also detected. Flag-PGK1 overexpressed HCT116 cells were transfected with exogenous Myc-PRMT1 **(C)**, shPRMT1 **(D)**, or treated with GSK3368715 **(E)** to detect the methylation changes of ADMA. **F**, **G** Methylation of arginine at R206 and R330 was detected by LC-MS. **H**, **I** Wild and mutant Flag-PGK1 (WT, R206K, and R330K) plasmids were overexpressed, and methylation changes of ADMA were detected by IP of Flag in HCT116 and HEK293T cells. The relative intensity of ADMA level were quantified by software Image J. The working concentration of GSK3368715 is 3.1 nM for 24 h.

Therefore, we explored the proteins that bind with PRMT1 by mass spectrometry (MS) after IP of Flag-PRMT1. Our MS data suggested that phosphoglycerate kinase 1 (PGK1), a key metabolic enzyme in the glycolysis pathway [31], binds to PRMT1 (Table S1). Co-immunoprecipitation assays (Co-IP) were performed by IP Flag-PGK1 after ectopic expression of Flag-PGK1 in HEK293T and HCT116 cells. The results showed that PRMT1 could directly bind with PGK1 in HEK293T and HCT116 cells (Fig. 2A, B). Interestingly, our IP assays also found that there were ADMA modifications on the PGK1 protein (Fig. 2A, B). What’s more, the ADMA level was increased in stable Flag-PGK1-overexpressing HCT116 cells when Myc-PRMT1 was exogenously transfected (Fig. 2C). The ADMA level of PGK1 was reduced when PRMT1 was knocked down in HCT116 cells stably overexpressing Flag-PGK1 (Fig. 2D). Moreover, the amount of PGK1 ADMA modification was also reduced in HCT116 cells after treatment with GSK3368715 (Fig. 2E). The above results strongly demonstrate that PRMT1 mediates PGK1 ADMA modification.

To further explore the detailed arginine methylation site of PRMT1 catalyzing PGK1 ADMA modification, we performed methylation mass spectrometry analysis of PGK1 after IP of Flag-PGK1 in HCT116 cells. The methylation mass spectrometry data showed that the R206 and R330 arginine sites of PGK1 can be methylated by modification (Fig. 2F, G). To determine the main site of PGK1-ADMA modification, we first constructed R206K and R330K PGK1 mutant plasmids (the mutant site cannot be methylated by PRMT1). Then, we carried out IP Flag-PGK1 assays after ectopic expression of wild-type (WT), R206K or R330K mutant Flag-PGK1 in HCT116 cells and HEK293T cells. Our results revealed that the amount of modified PGK1 ADMA was significantly decreased in the R206K mutant Flag-PGK1 group compared to the WT group (Fig. 2H, I). These results strongly indicate that PRMT1 mainly catalyzes PGK1-R206 ADMA modification in CRC cells.

### PRMT1-mediated meR206-PGK1 promotes colorectal cancer cell glycolysis by facilitating ERK-mediated PGK1-S203 phosphorylation

Post-translational modification of PGK1 (phosphorylation, O-GlcNAcylation, acetylation, and so on) at multiple sites can regulate its mitochondrial and nuclear translocation under certain conditions [32, 33]. For example, ERK-mediated phosphorylation of PGK1-S203 (pS203-PGK1) can promote mitochondrial localization of PGK1, and PGK1 translocated to mitochondria acts as a protein kinase, which phosphorylates pyruvate kinase (PDHK1), inhibits PDH complex (PDH)-dependent pyruvate utilization in mitochondria, weakens mitochondrial function, enhances glycolysis activity, and promotes proliferation and tumorigenesis [31].

**Fig. 3:**
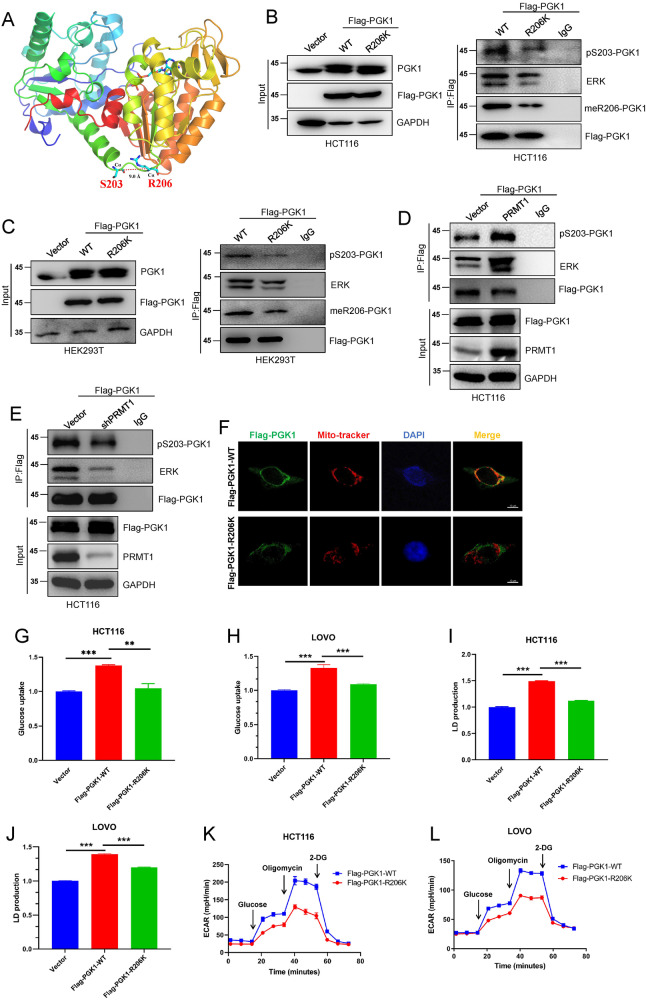
PRMT1-mediated meR206-PGK1 promotes colorectal cancer cell glycolysis by facilitating ERK-mediated PGK1-Ser203 phosphorylation. **A**–**C** The spatial positions of Serine 203 and arginine 206 in PGK1 were predicted **(A)**. The changes of pS203-PGK1 and meR206-PGK1 by IP of Flag, and the interaction between PGK1 and ERK were detected both in HCT116 cells **(B)** and HEK293T **(C)** cells when wild and mutant Flag-PGK1 (WT, R206K) were transiently overexpressed. **D**, **E** The amount of pS203-PGK1 and the PGK1-ERK interaction were detected by IP Flag-PGK1 after over-expressing PRMT1 **(D)** or silencing PRMT1 **(E)** in HCT116 cells, respectively. **F** Confocal detected the mitochondrial localization of PGK1 in wild and mutant Flag-PGK1 (WT, R206K) HCT116 cells. **G**–**L** Glucose uptake **(G, H)**, lactate production **(I, J)**, and extracellular acidification rates (ECAR) **(K, L)** were analyzed in stable overexpressed wild and mutant Flag-PGK1 (WT, R206K) HCT116 cells and LOVO cells. Data are represented as mean ± SEM of three independent experiments, and **p < 0.01, ***p < 0.001 (Student’s t-test).

Our previous studies have confirmed that there is crosstalk between protein arginine methylation and protein phosphorylation [15]. Through protein spatial structure analysis, we found that the S203 residue of PGK1 was very close to the R206 site (Fig. 3A). We speculated whether there is a crosstalk between R206 methylation and the S203 phosphorylation of these two sites, especially whether R206 methylation may have some effect on S203 phosphorylation. To verify this hypothesis, we first transiently transfected wild-type and mutant Flag-PGK1 (WT and R206K) plasmids into HCT116 and HER293T cells. Then, we detected the level of pS203-PGK1 changes in the WT and R206K groups after IP with Flag-PGK1. The results showed that both meR206-PGK1 and pS203-PGK1 expression were decreased in the R206K mutant group compared with the WT group (Fig. 3B, C). Furthermore, we also found that the ERK-PGK1 interaction ability was also strongly decreased after the R206-PGK1 mutation (Fig. 3B, C). Meanwhile, we detected the amount of pS203-PGK1 after over-expressing PRMT1 by IP Flag-PGK1 assays. Our data found that both the level of PGK1-ERK interaction and PGK1-S203 phosphorylation were increased (Fig. 3D). In contrast, we revealed that knockdown PRMT1 suppressed PGK1 binding with ERK and decreased the pS203-PGK1 level (Fig. 3E). Besides, we also found that ERK specific inhibitor SCH772984 dramatically repressed PGK1-ERK interaction and suppressed the increased amount of pS203-PGK1which mediated by ectopic expression of PRMT1 in HCT116 cells (Fig. S2).

Consistently, we detected PGK1 mitochondrial localization through confocal analysis of the above WT and R206K PGK1 overexpression in HCT116 cells. We found that PGK1 mitochondrial localization was significantly reduced in the Flag-PGK1-R206K group (Fig. 3F). Moreover, the glucose uptake and lactate production were also repressed in the Flag-PGK1-R206K group compared with the WT group in HCT116 cells and LOVO cells (Fig. 3G–J and Fig. S3A). In addition, the glycolytic capacity was also significantly impaired in the Flag-PGK1-R206K group compared with that in the Flag-PGK1-WT group (Fig. 3K, L). Above all, these results suggest that PRMT1-mediated meR206-PGK1 may increase ERK-mediated pS203-PGK and promote glycolysis in CRC cells.

### meR206-PGK1 inhibits the TCA cycle by promoting mitochondria-localized PGK1-mediated PDHK1 phosphorylation

Previous reports demonstrated that mitochondria-localized PGK1 can phosphorylate PDHK1 and inhibit PDHA1-dependent pyruvate utilization in mitochondria, which results in impaired mitochondrial function [31]. We further explored the effect of the mitochondrial localization of meR206-PGK1 on the oxygen consumption rate (OCR) in HCT116 cells mediated by PRMT1.

**Fig. 4:**
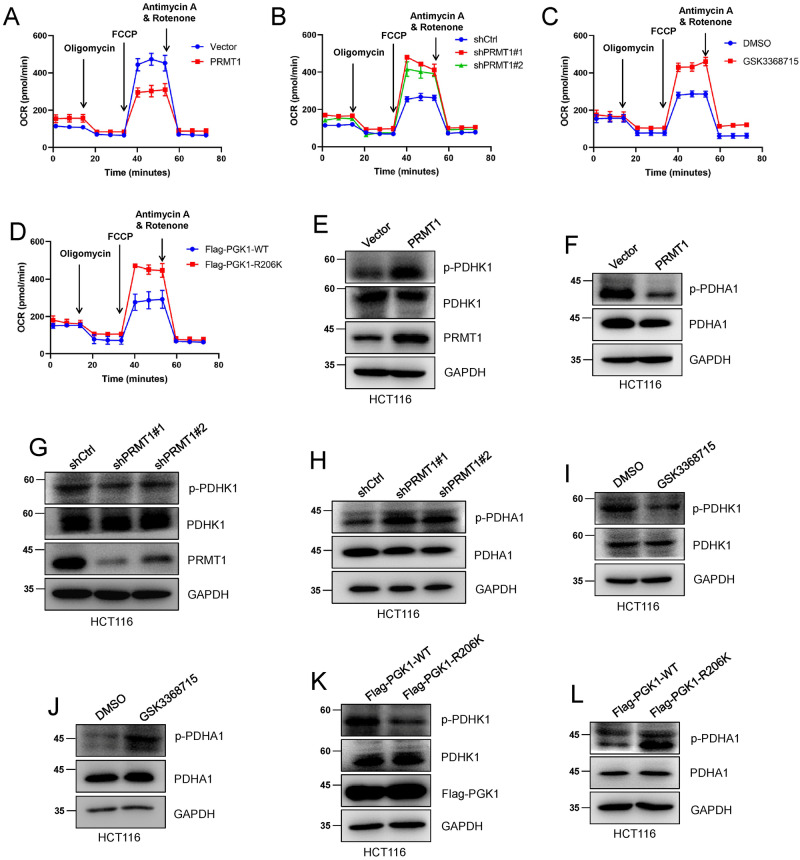
meR206-PGK1 inhibits the TCA cycle by promoting mitochondria localized PGK1 mediated PDHK1 phosphorylation. **A**–**D** Oxygen consumption rates (OCAR) were analyzed in HCT116 cells in which PRMT1were stable overexpressed **(A)**, knocked down **(B)** when HCT116 cells were treated with GSK3368715 **(C)**, and in stable overexpressed wild and mutant Flag-PGK1 (WT, R206K) HCT116 cells **(D)** by Seahorse Bioscience Extracellular Flux Analyzer. The working concentration of the drugs showed in **A**–**D** was Oligomycin (10 μM), FCCP (1 μM), and Antimycin A/Rotenone (50 μM). **E**–**L** PDHK1, PDHA1 and p-PDHK1, p-PDHA1 phosphorylation levels were detected in PRMT1 stable overexpressed **(E**, **F)**, knocked-down (**G**, **H**) HCT116 cells and when HCT116 cells were treated with GSK3368715 (3.1 nM, 48 hours) (**I**, **J**), and in stable overexpressed wild and mutant Flag-PGK1 (WT, R206K) HCT116 cells (**K**, **L**).

The results showed that the OCR was significantly decreased when PRMT1 was overexpressed in HCT116 cells (Fig. 4A), while the OCR was increased when PRMT1 was knocked down or treated with the PRMT1 inhibitor GSK3368715 in HCT116 cells (Fig. 4B, C). In addition, our data also showed that the OCR level was elevated in Flag-PGK1-R206K HCT116 cells compared with that in Flag-PGK1-WT HCT116 cells (Fig. 4D). Consistently, western blot data showed that PRMT1 overexpression increased the phosphorylation of PDHK1 and decreased the phosphorylation level of PDHA1 (Fig. 4E, F). The amount of p-PDHK1 decreased and p-PDHA1 increased when PRMT1 was knocked down or treated with GSK3368715 (Fig. 4G–J). Moreover, the stably expressed Flag-PGK1-R206K HCT116 cells showed low p-PDHK1 expression and high p-PDHA1 expression (Fig. 4K, L). These results suggest that PRMT1-mediated meR206-PGK1 inhibits the TCA cycle by promoting mitochondria-localized PGK1-mediated PDHK phosphorylation, inhibits PDHA1-dependent pyruvate utilization, and weakens mitochondrial function.

### PRMT1-mediated meR206-PGK1 promotes CRC cells proliferation and tumorigenesis

**Fig. 5:**
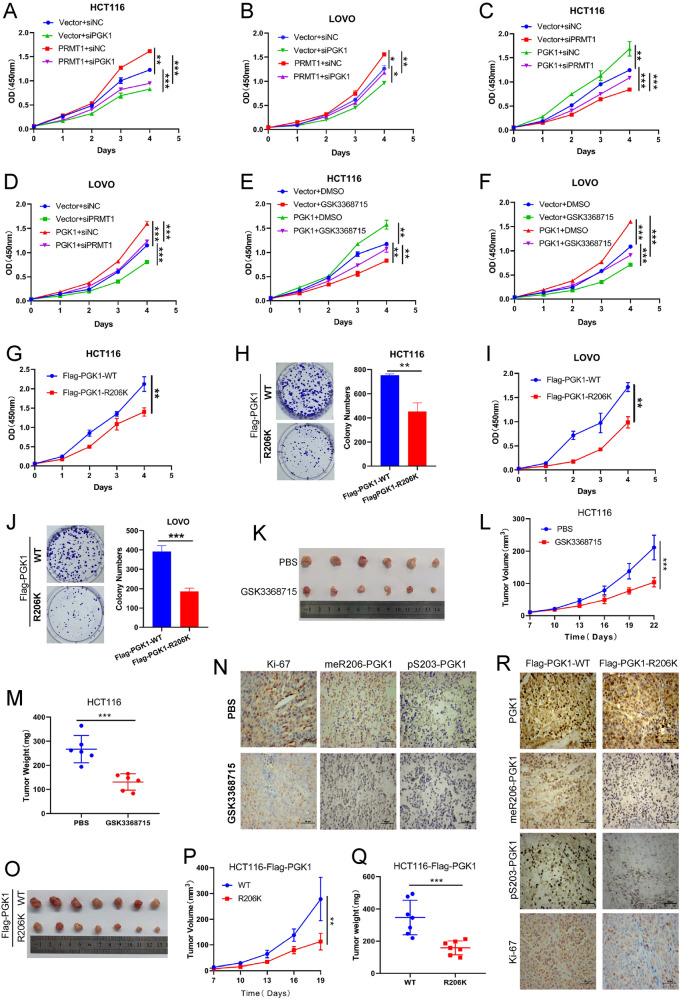
PRMT1-mediated meR206-PGK1 promotes colorectal cancer cell proliferation and tumorigenesis. **A, B** CCK-8 assays were used to assess the effect of PGK1 deficiency on cell proliferation in vector and PRMT1 stable overexpressed HCT116 cells (**A**) or LOVO cells **(B)**. **C**, **D** CCK-8 assays were used to assess the effect of PRMT1 deficiency on cell proliferation in Vector and PGK1 stable overexpressed HCT116 cells **(C)** or LOVO cells (**D**). **E**, **F** CCK-8 assays were used to assess the effect of GSK3368715 on cell proliferation in vector and PGK1 stable overexpressed HCT116 cells **(E)** or LOVO cells **(F)**. **G**, **J** CCK-8 assays and colony formation assays were used to assess the HCT116 cells (**G**, **H**) and the LOVO cells (**I**, **J**) proliferation abilities after stable over-expressing Flag-PGK1-WT or Flag-PGK1-R206K, respectively. **K**–**N** 4×10^6^ HCT116 cells and Matrigel (Corning; 1:1 ratio) were subcutaneously injected into the abdominal flanks of mice (*n* = 12). When the volume of the tumor reached about 50mm^3^, the mice were divided into two groups randomly: PBS and GSK3368715 (*n* = 6 for each group). The mice in the GSK3368715 group were treated with GSK3368715 100 mg/kg intraperitoneal everyday. Representative images **(K)**, tumor volumes **(L)** and tumor weights **(M)** of the two groups were shown. IHC detected Ki-67, meR206-PGK1, and pS203-PGK1 expression in xenograft tumors **(N)**. **O**–**R** 4×10^6^ stable overexpressed wild and mutant Flag-PGK1 (WT, R206K) HCT116 cells and Matrigel (Corning; 1:1 ratio) were subcutaneously injected into the abdominal flanks of mice (*n* = 7) to analyze the effect of meR206-PGK1 in tumorigenesis, representative images **(O)**, tumor volumes **(P)** and tumor weights **(Q)** of the two groups were shown. IHC detected PGK1, Ki-67, meR206-PGK1, and pS203-PGK1 expression in xenograft tumors **(R)**.

A variety of studies have revealed that cancer cells obtain energy mainly through glycolysis [34]. We further investigated whether PRMT1-mediated meR206-PGK1 affects CRC cells proliferation by CCK-8 assays. We first discovered that PRMT1 overexpression drastically increased the proliferation ability of HCT116 cells and LOVO cells, but silencing PGK1 weakened the increased proliferation ability caused by PRMT1 overexpression (Fig. 5A, B and Fig. S3B). At the same time, our colony formation assays also got the similar results **(**Fig. S4 A). Then, we also confirmed that the increased proliferation ability caused by PGK1 overexpression was attenuated when PRMT1 was knocked-down or after GSK3368715 treatment in PGK1 stably over-expressed HCT116 and LOVO cells by CCK-8 experiments (Fig. 5C–F). And our colony formation assays also disclosed that PRMT1 plays a key part in PGK1-mediated enhancing cell proliferation ability **(**Fig. S4B). In addition, both our colony formation assays and CCK-8 assays verified that the Flag-PGK1-R206K mutant group impaired cells proliferation ability compared with the Flag-PGK1-WT group in CRC cells (Fig. 5G–J). These results suggest that PRMT1-mediated meR206-PGK1 is important in promoting CRC cell proliferation in vitro.

To further evaluate the role of meR206-PGK1 in CRC tumorigenesis, we established xenograft models. HCT116 cells were subcutaneously transplanted into nude mice, and when the subcutaneous tumor grew to approximately 50mm^3^, mice were randomly divided into a control group (PBS), and a GSK3368715 treatment group (GSK3368715) according to tumor volume, with 6 mice in each group. The GSK3368715 treatment group was given GSK3368715 (100 mg/kg) intraperitoneally everyday for two consecutive weeks. As shown in our results, GSK3368715 treatment resulted in much smaller tumor volumes and tumor weights, and fewer Ki-67, meR206-PGK1, and pS203-PGK1 positive cells (Fig. 5K–N). What’s more, wild-type (Flag-PGK1-WT) and constructed Flag-PGK1-R206K mutant HCT116 cells were also subcutaneously transplanted into nude mice to detect the tumor formation. The Flag-PGK1-R206K group also showed smaller tumor volumes and tumor weights, and much fewer Ki-67, meR206-PGK1 and pS203-PGK1 positive cells compared with the counterparts in the Flag-PGK1-WT group (Fig. 5O–R). Taken together, these results further showed that PRMT1-mediated meR206-PGK1 promotes CRC cells proliferation and tumorigenesis in vitro and in vivo.

### PRMT1-mediated meR206-PGK1 positively correlates with colon cancer patient malignant progression and poor prognosis

**Fig. 6:**
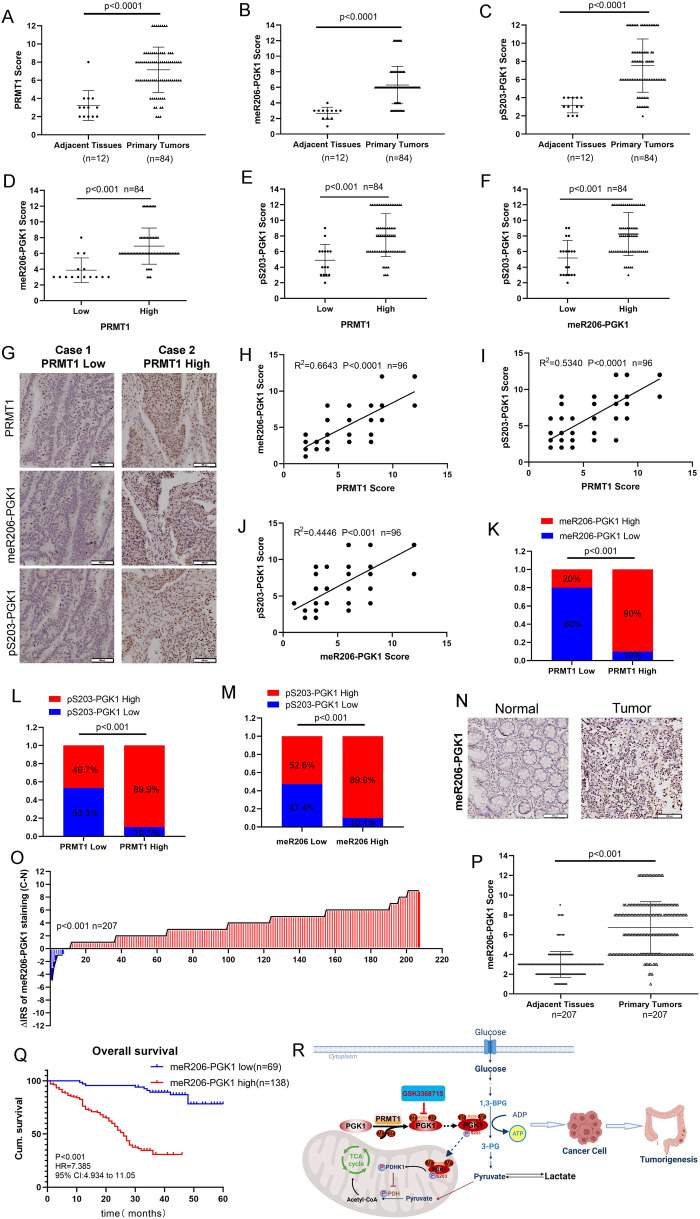
PRMT1-mediated meR206-PGK1 positive correlates with colon cancer patient malignant progression and poor prognosis. **A**–**G** IHC assays in colon cancer tissues were measured using anti-PRMT1, anti-meR206-PGK1 and anti-pS203-PGK1 antibodies (*n* = 96). Semi-quantitative scoring method (using a scale from 0 to 12) was used to quantify the scores of PRMT1, meR206-PGK1, and pS203-PGK1 IHC staining in adjacent tissues and primary tumors **(A–C)**. Analyzing the relevant meR206-PRMT1**(D)**, pS203-PRMT1 **(E)** expression in PRMT1-low cases and PRMT1-high cases; analyzing the relevant pS203-PGK1 expression in meR206-PGK1-low cases and meR206-PGK1-high cases **(F)** by Student’s t-test. Representative images of PRMT1, meR206-PGK1 and pS203-PGK1 expressions in PRMT1-high case and PRMT1-low case were presented **(G)**. **H**–**M** Correlation between PRMT1 and meR206-PGK1 expression **(H)**, PRMT1 and pS203-PGK1 expression **(I)**, meR206-PGK1 and pS203-PGK1 **(J)** were examined by Pearson’s correlation coefficient test, respectively (*n* = 96). Correlation between PRMT1 and meR206-PGK1 expression **(K)**, PRMT1 and pS203-PGK1 expression **(L)**, pS203-PGK1 and meR206-PGK1 **(M)** were examined by Fisher’s exact test, respectively (*n* = 96). **N**–**Q** Representative IHC images of meR206-PGK1 protein expression in normal and tumor tissues in CRC patients (*n* = 207) **(N)**. Staining intensities of meR206-PGK1 in colorectal carcinoma tissues compared with paired adjacent non-cancerous tissue. C, colorectal carcinoma tissues; N, paired adjacent non-cancerous tissues. (*n* = 207) **(O)**. IHC assays in colon cancer tissues (*n* = 207) were measured using anti-meR206-PGK1 antibodies. Semi-quantitative scoring method (using a scale from 0 to 12) was used to quantify the scores of meR206-PGK1 **(P)**. Kaplan–Meier survival curves depicting overall survival (*n* = 207, *p* < 0.001) stratified by meR206-PGK1 protein expression levels in CRC tissues **(Q)**. **R** A cartoon summarizing our findings. PRMT1 catalyzed the methylation of arginine at R206 of PGK1 protein, increased the phosphorylation level at S203 of PGK1 in colorectal cancer cells, leading to the inhibition of the mitochondrial function, and enhanced the ability of glycolysis, which accelerated the occurrence and development of colorectal cancer. Targeting glycolysis with PRMT1 inhibitors in the treatment of colorectal cancer has great clinical potential.

CRC TMA slides including 12 cases of adjacent tissues and 84 cases of primary tumors were used to assess the expression of PRMT1, meR206-PGK1, and pS203-PGK1 to evaluate the clinical significance of our studies. We found that compared with adjacent tissues, primary tumors had higher PRMT1, meR206-PGK1, and pS203-PGK1 expression (Fig. 6A–C). CRC patients with high PRMT1 expression were also had highly expressed meR206-PGK1 (Fig. 6D) and pS203-PGK1 (Fig. 6E). What’s more, we also found that high meR206-PGK1 expression was also associated with high pS203-PGK1 expression in CRC tissues (Fig. 6F). A representative image is shown in Fig. 6G.

We further analyzed the expression correlation among PRMT1, meR206-PGK1, and pS203-PGK1 in CRC patients. The results showed that PRMT1 was positively correlated with meR206-PGK1 and pS203-PGK1 (Fig. 6H, I). We also confirmed that meR206-PGK1 was positively correlated with pS203-PGK1 (Fig. 6J). Moreover, we found that high PRMT1 expression was positively correlated with both high meR206-PGK1 expression, and high pS203-PGK1 expression (Fig. 6K, L), and high expression of meR206-PGK1 was positively correlated with high pS203-PGK1 expression (Fig. 6M).

We further detected meR206-PGK1 expression in 207 CRC patient samples. meR206-PGK1 expression was significantly increased in CRC primary tumors compared with normal tissues **(**Fig. 6N–P**)**. The correlation between meR206-PGK1 expression and clinicopathologic characteristics in CRC tissue samples was analyzed **(**Table 1, Table S2**)**. The expression of meR206-PGK1 was dramatically positively correlated with tumor diameter and TNM stage (*p* < 0.001). Kaplan–Meier survival analysis also revealed that high meR206-PGK1 levels were correlated with poor overall survival in CRC patients **(**Fig. 6Q**)**. Overall, these clinical data indicated that meR206-PGK1 has great potential as a therapeutic and prognostic target in CRC.

**Table 1:** Relationship between meR206-PGK1 expression and clinicopathological features in 207 CRC patients.

Variables	meR206-PGK1 expression (*n* = 207 cases)		
	Low (%)	High (%)	*P*
Age (years)			0.164
≤60	34 (38.6)	54 (61.4)	
>60	35 (29.4)	84 (70.6)	
Gender			0.921
Males	39 (33.1)	79 (66.9)	
Females	30 (33.7)	59 (66.3)	
Depth of invasion			0.055
T1/T2	20 (45.5)	24 (54.5)	
T3/T4	49 (30.1)	114 (69.9)	
Lymph node metastasis			<0.001
N0	52 (43.7)	67 (56.3)	
N1/N2/N3	17 (19.3)	71 (80.7)	
Distant metastasis			0.522
M0	68 (33.7)	134 (66.3)	
M1	1 (20.0)	4 (80.0)	
TNM stage			<0.001
I/II	53 (46.1)	62 (53.9)	
III/ IV	16 (17.3)	76 (82.6)	
Tumor diameter			<0.001
<5 cm	63 (45.3)	76 (54.7)	
≥5 cm	6 (8.8)	62 (91.2)	
Differentiation			0.059
Poor	8 (20.5)	31 (79.5)	
Moderate/high	61 (36.3)	107 (63.7)	

^*^P values are from χ^2^ test.

## Discussion

In this study, we demonstrated that PRMT1 catalyzed the methylation of arginine at the R206 of PGK1 protein, and increased ERK-mediated phosphorylation at PGK1-S203, leading to enhanced glycolytic activity and CRC tumorigenesis **(**Fig. 6R**)**. We also verified that meR206-PGK1 expression is positively correlated with PRMT1 and pS203-PGK1 in CRC tissue samples. meR206-PGK1 is increased in CRC tissues and is positively correlated with tumor diameter and poor prognosis.

PRMT1 participates in the asymmetric dimethylation modification of arginine 3 on histone H4, necrotic apoptosis, and immune regulation in CRC [35–37]. Here, we identified a novel function of PRMT1 in abnormal glycolysis in CRC. PRMT1 catalyzes the methylation of arginine at R206 of the PGK1 protein, leading to enhanced glycolysis. It should be noted that glycolysis is a complex enzymatic reaction, and whether other enzyme components can also be methylated should be further confirmed. What’s more, it has been reported that lipogenic enzymes are significantly upregulated in CRC, FASN gene expression is prognostically detrimental [38], and lipid droplet accumulation also contributes to chemotherapy resistance in CRC [39]. The role of PRMT1 in enhanced lipid metabolism is unknown, and whether there is crosstalk between abnormal glycolysis and lipid metabolism mediated by PRMT1 still needs to be studied.

Nuclear PGK1 preferentially drives cell metastasis via mitochondrial oxidative phosphorylation induction, whereas cytoplasmic PGK1 preferentially supports proliferation by functioning as a glycolytic enzyme [40]. PGK1 can be dynamically modified with O-linked N-acetylglucosamine (O-GlcNAc) at threonine 255 (T255), which induces PGK1 translocation into mitochondria to inhibit the PDH complex and enhance glycolysis [33]. Moreover, PGK1 also functions as a protein kinase intermolecularly auto-phosphorylating itself at Y324 for activation, leading to enhanced glycolysis [41]. As an arginine methyltransferase, PRMT1 regulates the expression of target genes by catalyzing arginine methylation modification of target proteins, and many substrates of PRMT1 have been identified [42–44]. We found that PRMT1 mediated meR206-PGK1 and increased ERK-mediated phosphorylation at PGK1-S203. The effect of meR206 on the modification of PGK1 at other sites still needs to be studied.

Specific inhibitors developed for protein methyltransferase can significantly inhibit the growth of tumor cells. In this study, we revealed the inhibitory effect of arginine methyltransferase on CRC cell glycolysis, which also showed anti-tumor effects in vivo. Targeting glycolysis with arginine methyltransferase inhibitors in the treatment of colorectal cancer shows great clinical potential. However, whether there will be adverse reactions in clinical applications still needs more research.

In summary, we propose a potential mechanism by which PRMT1 enhances CRC glycolysis and tumorigenesis by mediating PGK1 arginine dimethylation modification at R206, which promotes ERK-mediated PGK1 phosphorylation at S203 sites, and PRMT1, meR206-PGK1, and pS203-PGK1 may be reliable combinational biomarkers for CRC prognosis. These biomarkers have great application prospects for protein arginine methyltransferase inhibitors in CRC treatments.

### Supplementary information

Supplementary Materials and Methods.

Figure S1

Figure S2

Figure S3

Figure S4

Supplementary Table 1

Supplementary Table2

Original Data File

reproducibility checklist

## Data Availability

The data supporting the findings of this study are available from the corresponding author upon reasonable request.
