# Supplementary material for: PRMT1-mediated PGK1 arginine methylation promotes colorectal cancer glycolysis and tumorigenesis
Source: Cell Death Dis. 2024 Feb 24;15(2):170. doi: 10.1038/s41419-024-06544-6 (PMC10894231; doi:10.1038/s41419-024-06544-6)
Supplement: Supplementary file 1 — Supplementary Materials and Methods. [file 41419_2024_6544_MOESM1_ESM.docx]

**PRMT1-mediated PGK1 arginine methylation promotes colorectal cancer glycolysis and tumorigenesis**

**Supplementary Materials**

The sequences of siRNAs used for gene silencing

| siRNAs | Target RNA Sequence |
| --- | --- |
| si-NC | AUGGAAGAUGAUCUGGUGA |
| si-PRMT1#1 | GGACAUGACAUCCAAAGAUUA |
| si-PRMT1#2 | GUGUUCCAGUAUCUCUGAUUA |
| si-PGK1#1 | GCUUCUGGGAACAAGGUUAAA |
| si-PGK1#2 | CUGACAAGUUUGAUGAGAAUG |

**Supplementary Methods**

**1. Assessment of IHC**

Three pathologists assessed separately the TMAs under blinded experimental conditions and all differences that arise were resolved by discussion. The staining scores of PRMT1, meR206-PGK1, and pS203-PGK1 were evaluated by combining the percentage of cells with the staining intensity and being dependent on the IRS (immunoreactivity score, IRS). The intensity of PRMT1, meR206-PGK1, and pS203-PGK1 immunostaining were scored as 0–3 (0, negative; 1, weak; 2, moderate; 3, strong); the percentage of immunoreactivity cells was graded as 1 (0–25%), 2 (26–50%), 3 (51–75%), and 4 (76–100%). Relied on the IRS, the level of PRMT1 and meR206-PGK1 expression was categorized as low (IRS: 0–4) and high (IRS: 4–12) expression.

**2. Seahorse assays**

Cellular glycolysis was monitored using Seahorse Bioscience Extracellular Flux Analyzer (XF96, Seahorse Bioscience Inc., North Billerica, MA, USA) by measuring extracellular acidification rate (ECAR) in real-time as described in the manufacturer’s instructions. In brief, the over-expressed, knocked-down of PRMT1 or GSK3368715 treated HCT116 cells were plated in the wells of 96-well plates (3×10^4^ cells/well for ECAR; (2.5×10^4^ cells/well for OCAR XF96 plates; Seahorse Bioscience) and incubated overnight at 37°C. The next day, the medium was changed to XF assay medium and loaded with (glucose, oligomycin, and 2-DG for ECAR), and (oligomycin, FCCP, and Antimycin A & Rotenone for OCAR) respectively, according to the manufacturer’s recommendation. All the tests were performed on the XFe96 bioanalyzer.

**3. Glucose uptake, and lactate production**

Cells were plated in six-well plates for 48 hours after the over-expressed, knocked-down of PRMT1 or GSK3368715 treatment. Culture medium was collected and subjected to the glucose assay kit (Shanghai Rongsheng Biotech, Shanghai, China) and lactate assay kit (Shanghai Rongsheng Biotech, Shanghai, China) to measure glucose uptake and lactate production following the manufacturer’s instructions.

**4. Cell proliferation and colony formation assays**

CCK-8 assays were carried out to determine the function of PRMT1 and PGK1 on cell proliferation. In brief, after the knockdown of PGK1 in the PRMT1-overexpressed HCT116 cells, or after the knockdown of PRMT1 in the PGK1-overexpressed HCT116 cells, or HCT116 cells treated with GSK3368175 (3.1nM) for 24h, or the Flag-PGK1-WT and Flag-PGK1-R206K HCT116 cells were seeded in each well of 96 well plates (3×10^3^ cells/well), and CCK-8 solution was added 24, 48, 72, and 96h after placing. Cells were incubated at 37 °C for 1 h after 10μl CCK-8 solution was added. For colony formation assay, 1 × 103 cells were cultured in 60mm plate at 37 °C for 14 days. Visible colonies were washed twice with phosphate-buffered saline (PBS), fixed and stained with 4% paraformaldehyde and crystal violet, respectively. The number of colonies was counted visually.

**5. Generation of stable cells using lentivirus**

PRMT1, Flag-PGK1-WT and Flag-PGK1-R206K cDNA were cloned to the pCDH1-CMV-MSC-EF1-Puro vector. Lentiviruses were produced by co-transfecting HEK293T cells with one of the expression plasmids and the packaging plasmids (psPAX2 and pMD2.G). The supernatants were collected after 48 hours, and filtered through 0.45μm filters (Millipore, Temecula, CA, USA), then concentrated using Amicon Ultra centrifugal filters (Millipore 100KD MWCO). The concentrated viruses were used to infect HCT116 cells. Stable transfection cell lines were selected with 2mg/ml puromycin for 15 days.

**Supplementary Figure Legends**

**Supplement Figures:**

**Fig. S1 PRMT1 expression confirming after over-expressing of knocking down PRMT1. A-B** Western blots detecting PRMT1 expression after ectopic expression of PRMT1 in HCT116 cells and DLD1 cells. **C-D** Western blots detecting PRMT1 expression after knockdown PRMT1 in HCT116 cells and DLD1 cells.

**Fig. S2** Western blots detecting PGK1-ERK interaction and pS203-PGK1 expression by IP Flag-PGK1 in Flag-PGK1-HCT116 cells after treated with ERK specific inhibitor SCH772984 (10nM, 48 hours).

**Fig. S3 A** Western blots detecting Flag-PGK1 expression after ectopic expression of wild type PGK1 or R206K mutant PGK1 in LOVO cells. **B** Western blots detecting PRMT1 expression after ectopic expression of PRMT1 in LOVO cells.

**Fig. S4 A** Colony formation assays were used to assess the effect of PGK1 deficiency on cell proliferation in vector and PRMT1 stable overexpressed HCT116 cells. **B** Colony formation assays were used to assess the effect of PRMT1 deficiency on cell proliferation in Vector and PGK1 stable overexpressed HCT116 cells.
