# Supplementary figures and images for: PRMT1-mediated PGK1 arginine methylation promotes colorectal cancer glycolysis and tumorigenesis
Source: Cell Death Dis. 2024 Feb 24;15(2):170. doi: 10.1038/s41419-024-06544-6 (PMC10894231; doi:10.1038/s41419-024-06544-6)

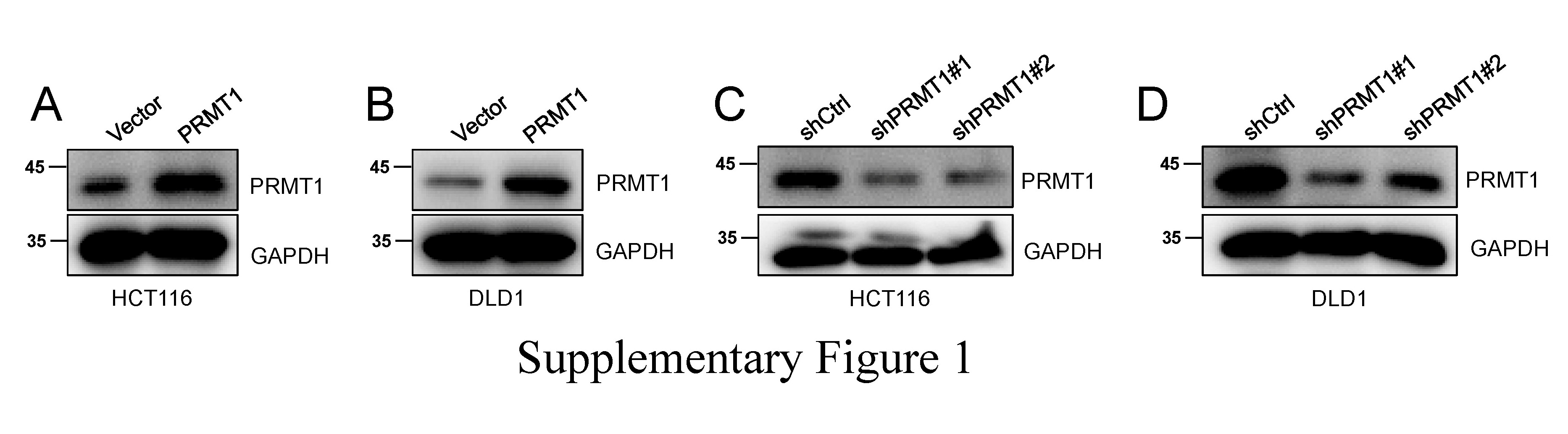

Supplement: Supplementary file 2 — Figure S1 [file 41419_2024_6544_MOESM2_ESM.jpg]

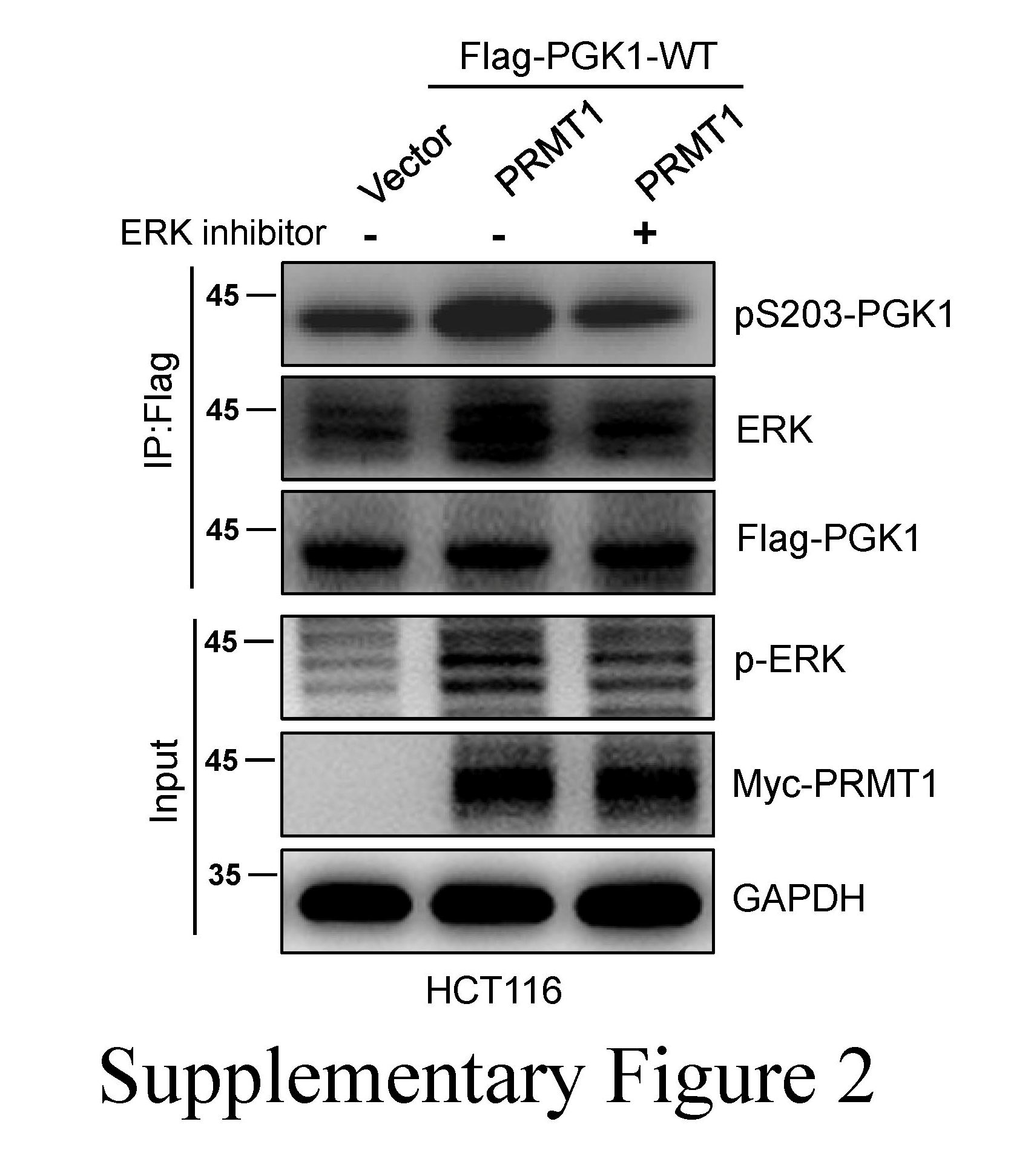

Supplement: Supplementary file 3 — Figure S2 [file 41419_2024_6544_MOESM3_ESM.jpg]

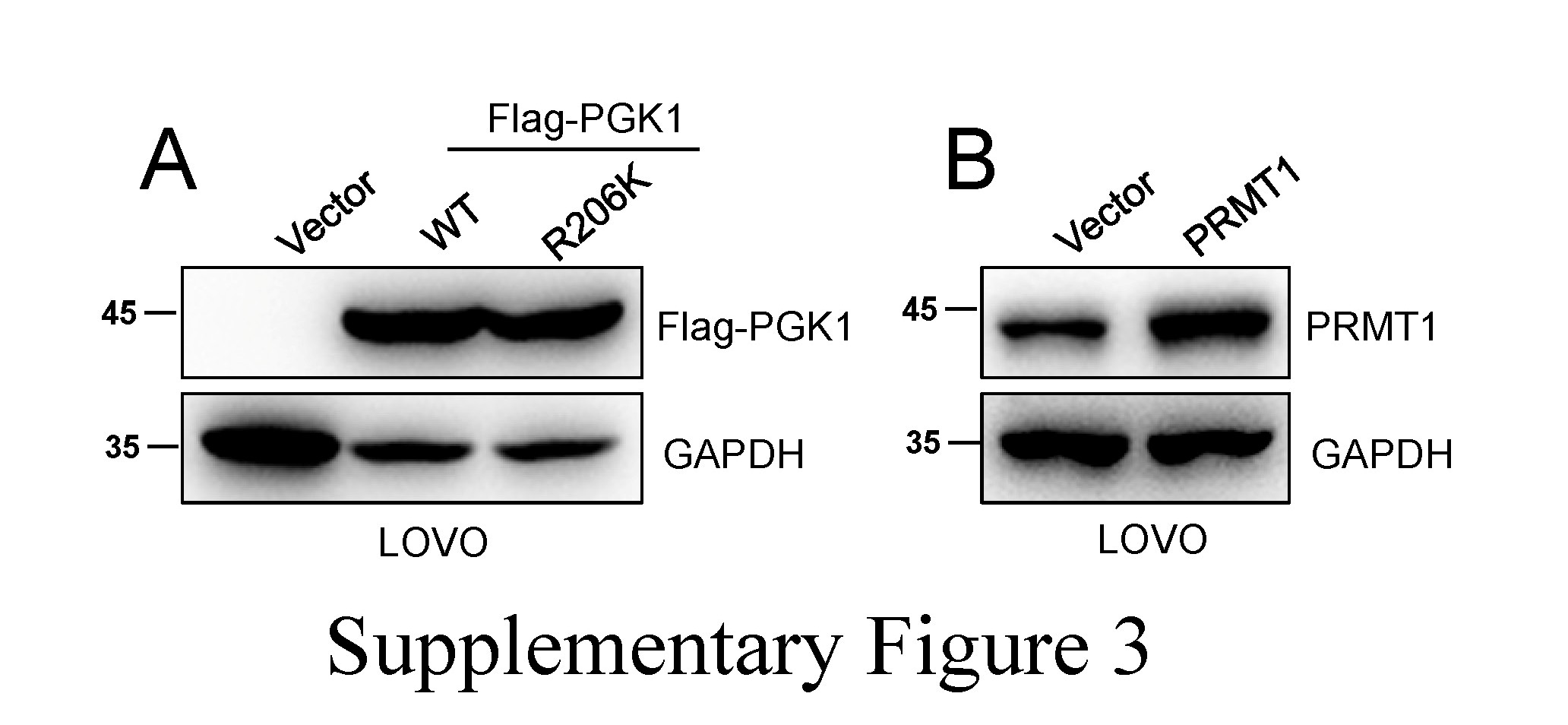

Supplement: Supplementary file 4 — Figure S3 [file 41419_2024_6544_MOESM4_ESM.jpg]

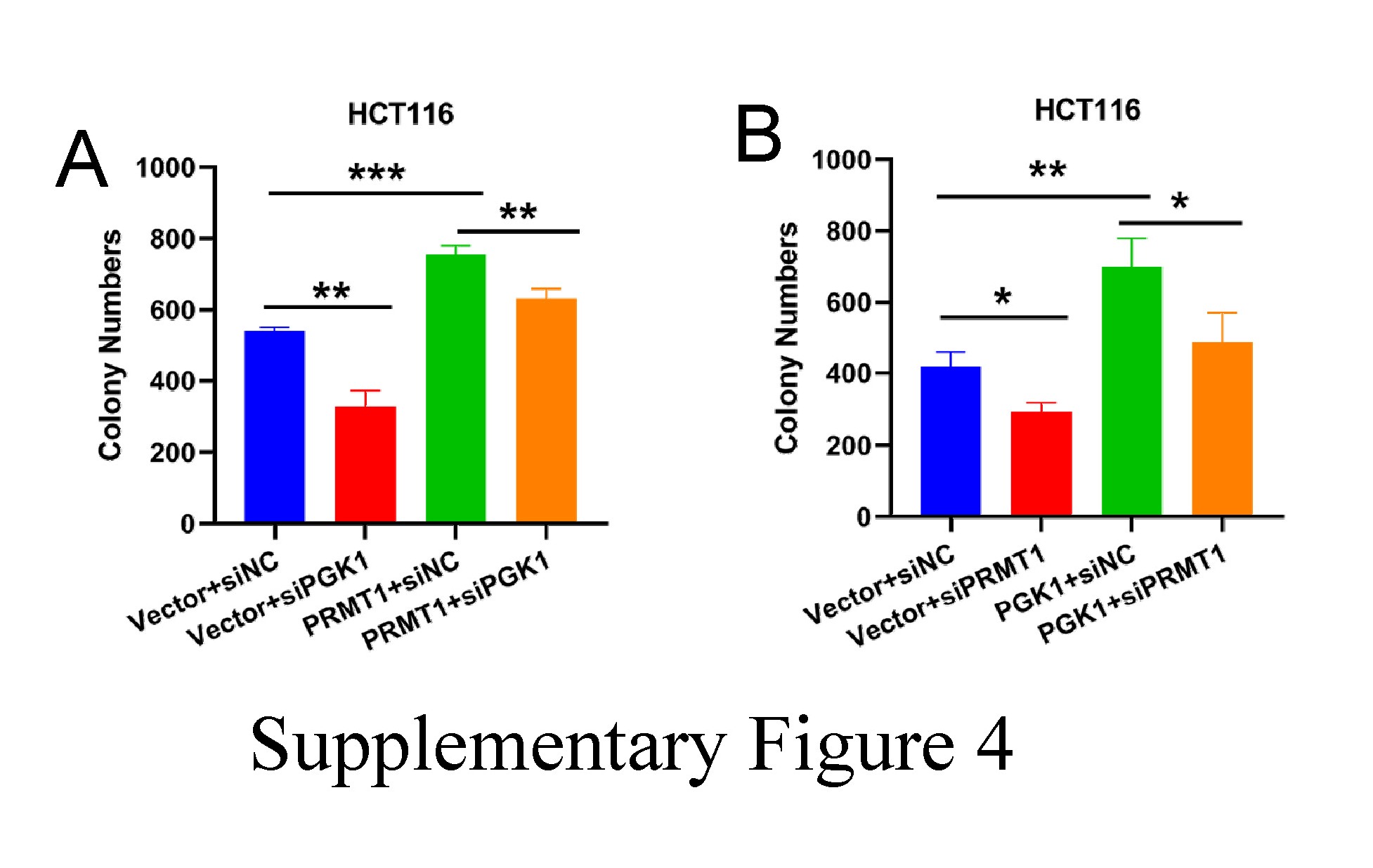

Supplement: Supplementary file 5 — Figure S4 [file 41419_2024_6544_MOESM5_ESM.jpg]
