# Supplementary material for: PRMT1-mediated PGK1 arginine methylation promotes colorectal cancer glycolysis and tumorigenesis
Source: Cell Death Dis. 2024 Feb 24;15(2):170. doi: 10.1038/s41419-024-06544-6 (PMC10894231; doi:10.1038/s41419-024-06544-6)
Supplement: Supplementary file 7 — Supplementary Table2 [file 41419_2024_6544_MOESM7_ESM.docx]

**Supplementary Table 2.** Multivariate Cox regression analysis of the 5-year overall survival of 207 CRC patients.

| **Variable*** | **Overall survival** | | |
| --- | --- | --- | --- |
|  | **Hazard ratio** | **95％CI^†^** | **P*** |
| meR206-PGK1 | 7.385 | 4.985-22.554 | <0.001 |
| Age | 1.159 | 0.752-1.785 | 0.503 |
| Gender | 1.573 | 1.017-2.431 | 0.042 |
| Tumor size | 2.188 | 1.447-3.309 | <0.001 |
| TNM stage | 1.097 | 0.731-1.645 | 0.655 |
| Distant metastasis | 2.281 | 0.720-7.229 | 0.161 |
| Lymph node metastasis | 1.021 | 0.678-1.537 | 0.921 |
| Depth of invasion | 1.494 | 0.883-2.529 | 0.135 |

* *P* values are from Log-rank test. † CI: confidence interval.

^*^Coding of variables: meR206-PGK1 was coded as 1 (low), and 2 (high). Gender was coded as 1 (Male), and 2 (Female). Age was coded as 1 (≤60 years), and 2 (>60 years). Tumor size was coded as 1 (≤5 cm), and 2 (>5 cm). TNM stage was coded as 1 (I/II), and 2 (III/ IV). Distance metastasis was coded as 1 (M0), and 2 (M1). Lymph node metastasis was coded as 1 (N0), and 2 (N1/N2/N3). Depth of invasion was coded as 1 (T1/T2), and 2 (T3/T4). ^†^CI: confidence interval.
