# Supplementary material for: PRMT1-mediated PGK1 arginine methylation promotes colorectal cancer glycolysis and tumorigenesis
Source: Cell Death Dis. 2024 Feb 24;15(2):170. doi: 10.1038/s41419-024-06544-6 (PMC10894231; doi:10.1038/s41419-024-06544-6)
Supplement: Supplementary file 8 — Original Data File [file 41419_2024_6544_MOESM8_ESM.pdf]

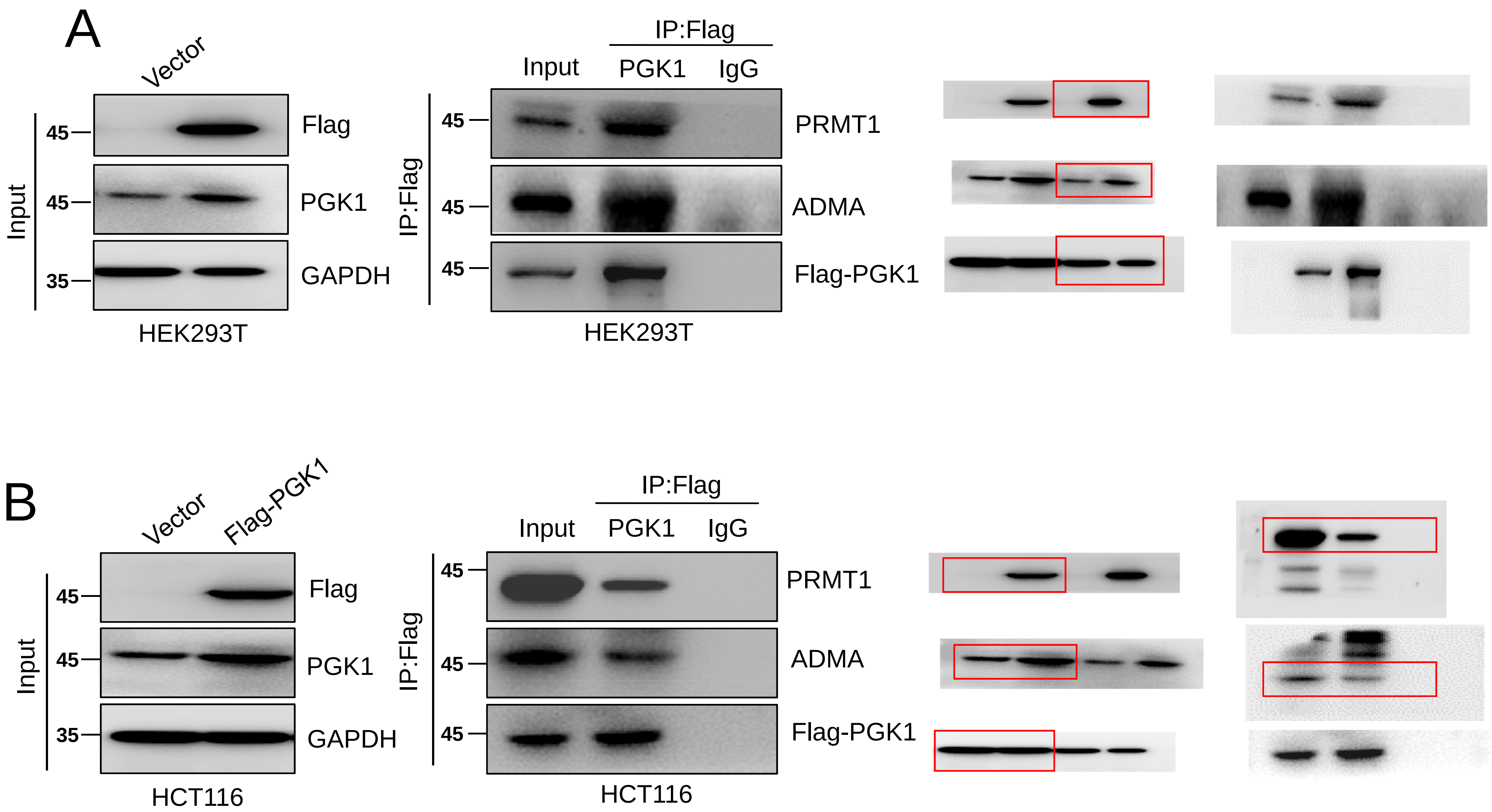
A
Vector
Input
45
45
35
Flag
PGK1
GAPDH
HEK293T
IP:Flag
Input
PGK1
IgG
IP:Flag
45
45
45
PRMT1
ADMA
Flag-PGK1
HEK293T
B
Vector
Flag-PGK1
Input
45
45
35
Flag
PGK1
GAPDH
HCT116
IP:Flag
Input
PGK1
IgG
IP:Flag
45
45
45
PRMT1
ADMA
Flag-PGK1
HCT116

Figure 2

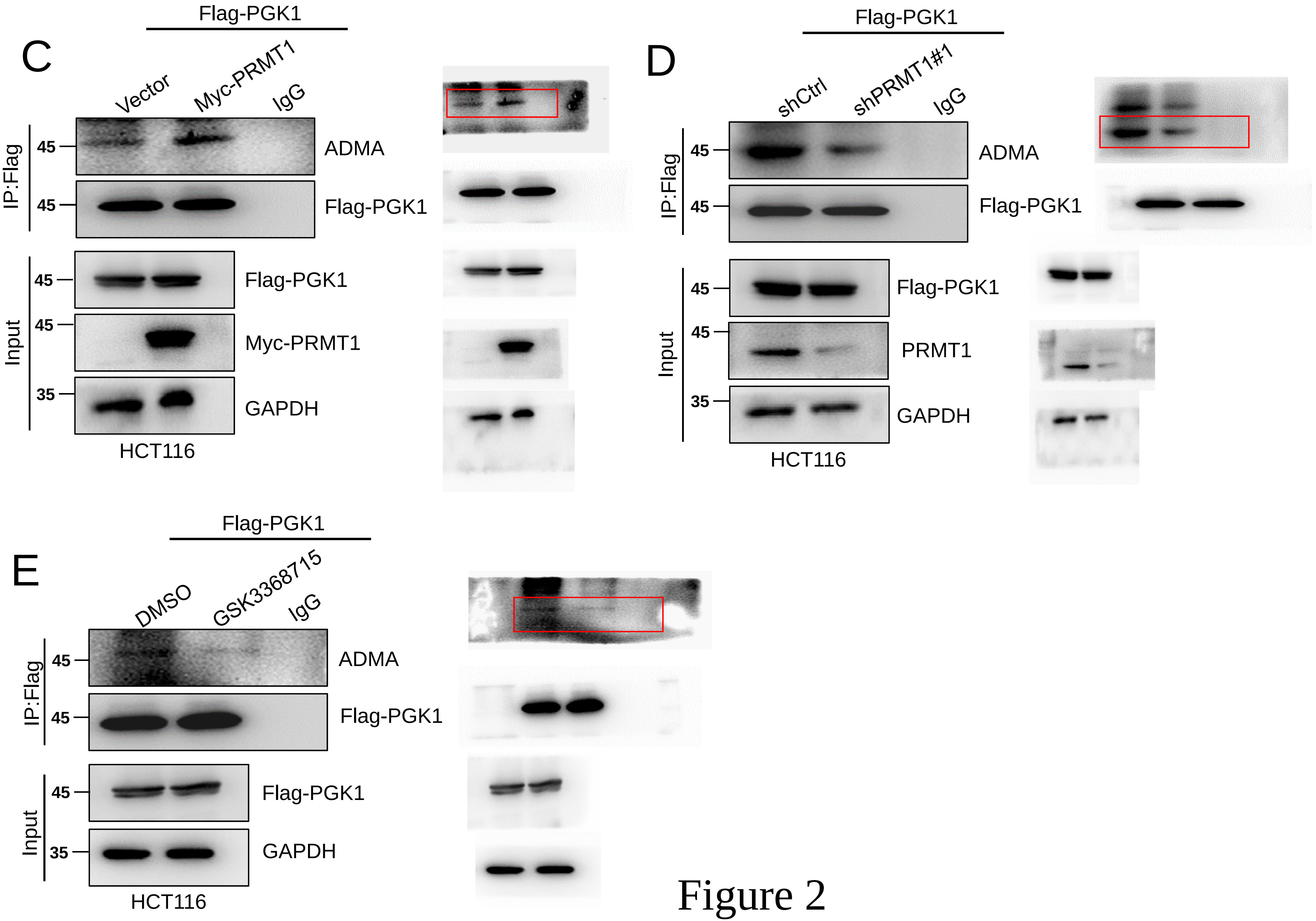

C
Flag-PGK1
Vector
Myc-PRMT1
IgG
IP:Flag
45
ADMA
45
Flag-PGK1
Input
45
Flag-PGK1
45
Myc-PRMT1
35
GAPDH
HCT116
D
Flag-PGK1
shCtrl
shPRMT1#1
IgG
IP:Flag
45
ADMA
45
Flag-PGK1
Input
45
Flag-PGK1
45
PRMT1
35
GAPDH
HCT116
E
Flag-PGK1
DMSO
GSK3368715
IgG
IP:Flag
45
ADMA
45
Flag-PGK1
Input
45
Flag-PGK1
35
GAPDH
HCT116

Figure 2

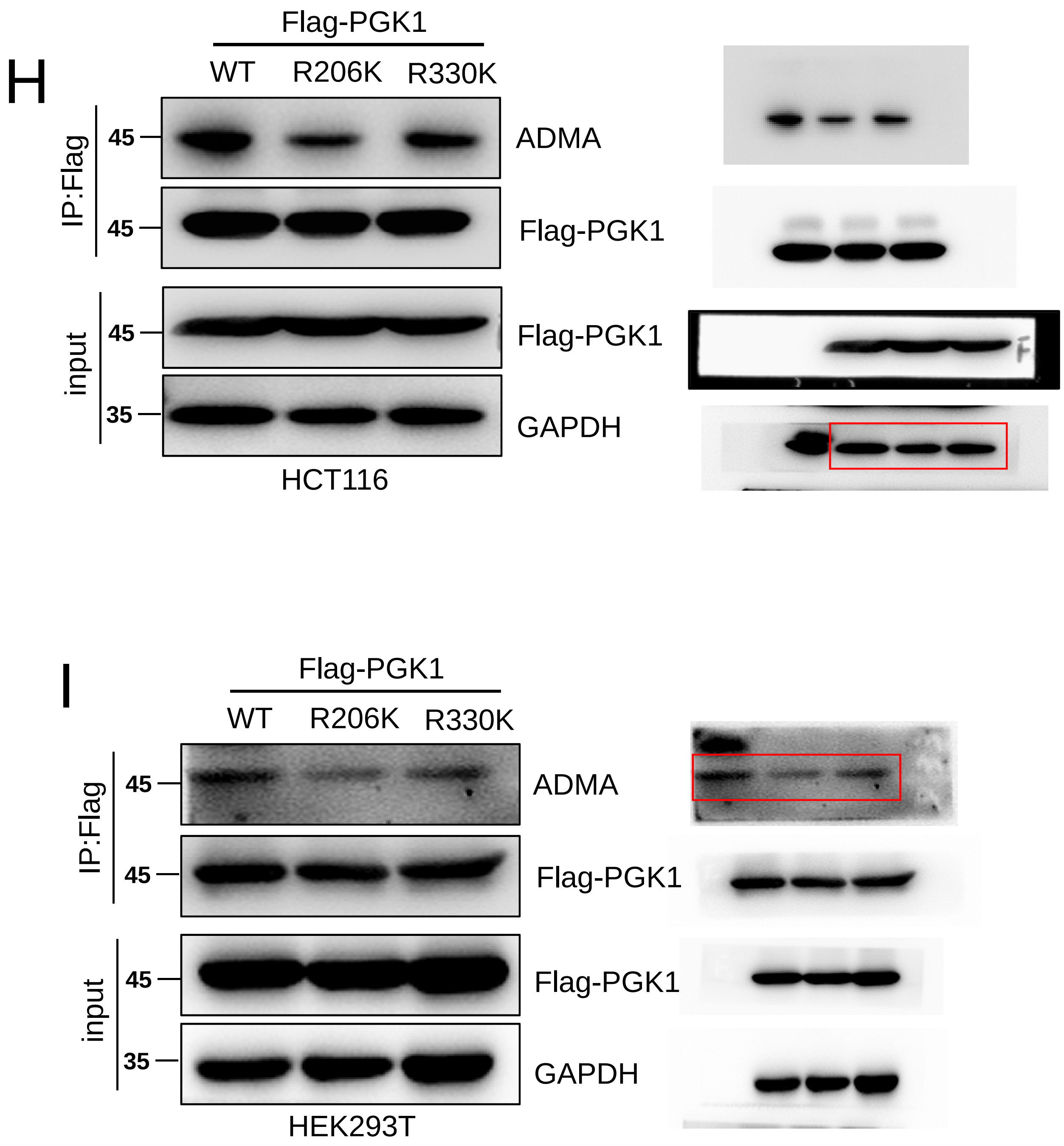
H
Flag-PGK1
WT
R206K
R330K
IP:Flag
45
ADMA
45
Flag-PGK1
input
45
Flag-PGK1
35
GAPDH
HCT116
I
Flag-PGK1
WT
R206K
R330K
IP:Flag
45
ADMA
45
Flag-PGK1
input
45
Flag-PGK1
35
GAPDH
HEK293T

Figure 2

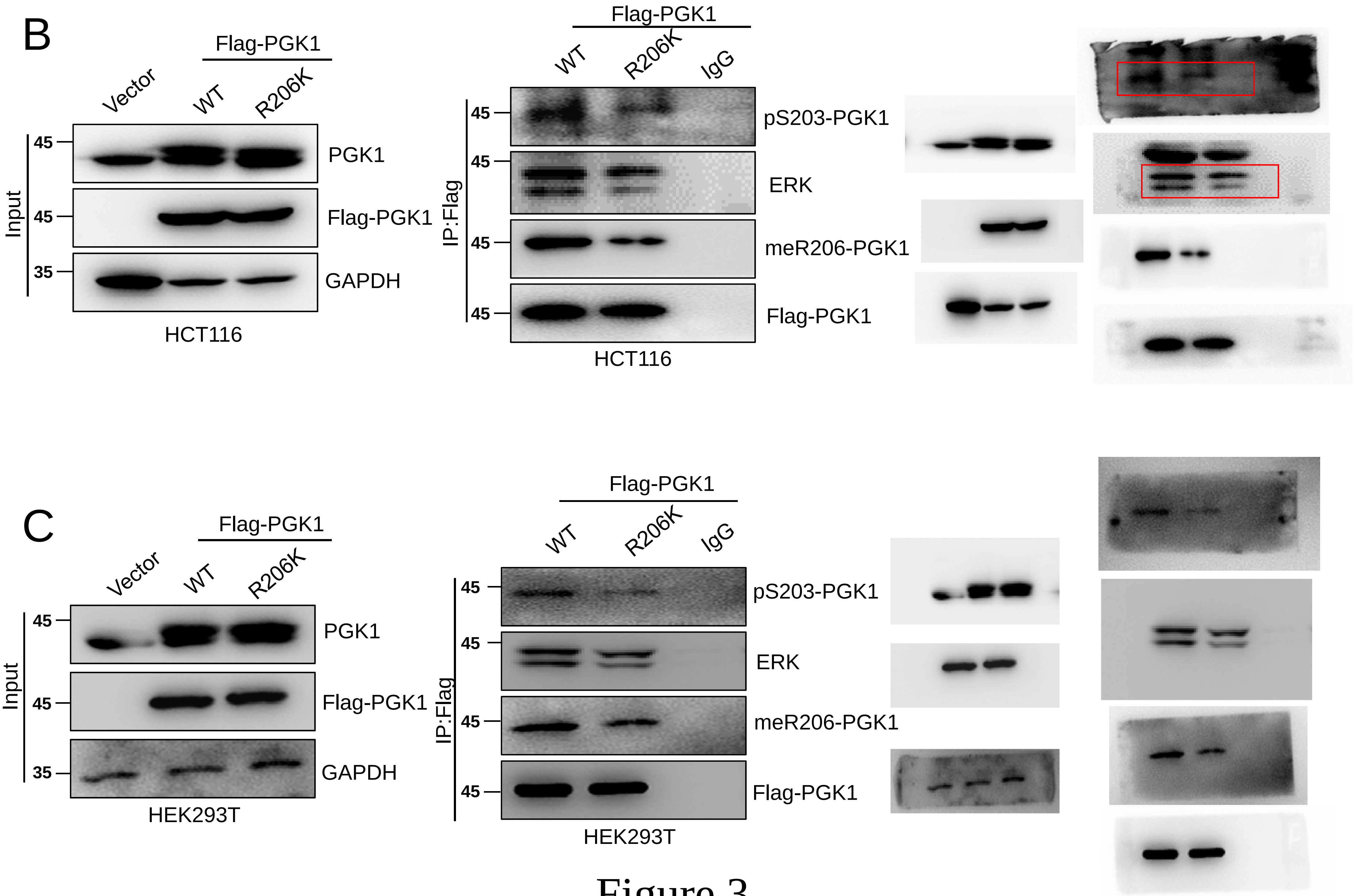
B
Flag-PGK1
Vector
WT
R206K
45
PGK1
45
Flag-PGK1
35
GAPDH
Input
HCT116
Flag-PGK1
WT
R206K
IgG
45
pS203-PGK1
45
ERK
45
meR206-PGK1
45
Flag-PGK1
IP:Flag
HCT116
C
Flag-PGK1
Vector
WT
R206K
45
PGK1
45
Flag-PGK1
35
GAPDH
Input
HEK293T
Flag-PGK1
WT
R206K
IgG
45
pS203-PGK1
45
ERK
45
meR206-PGK1
45
Flag-PGK1
IP:Flag
HEK293T

Figure 3

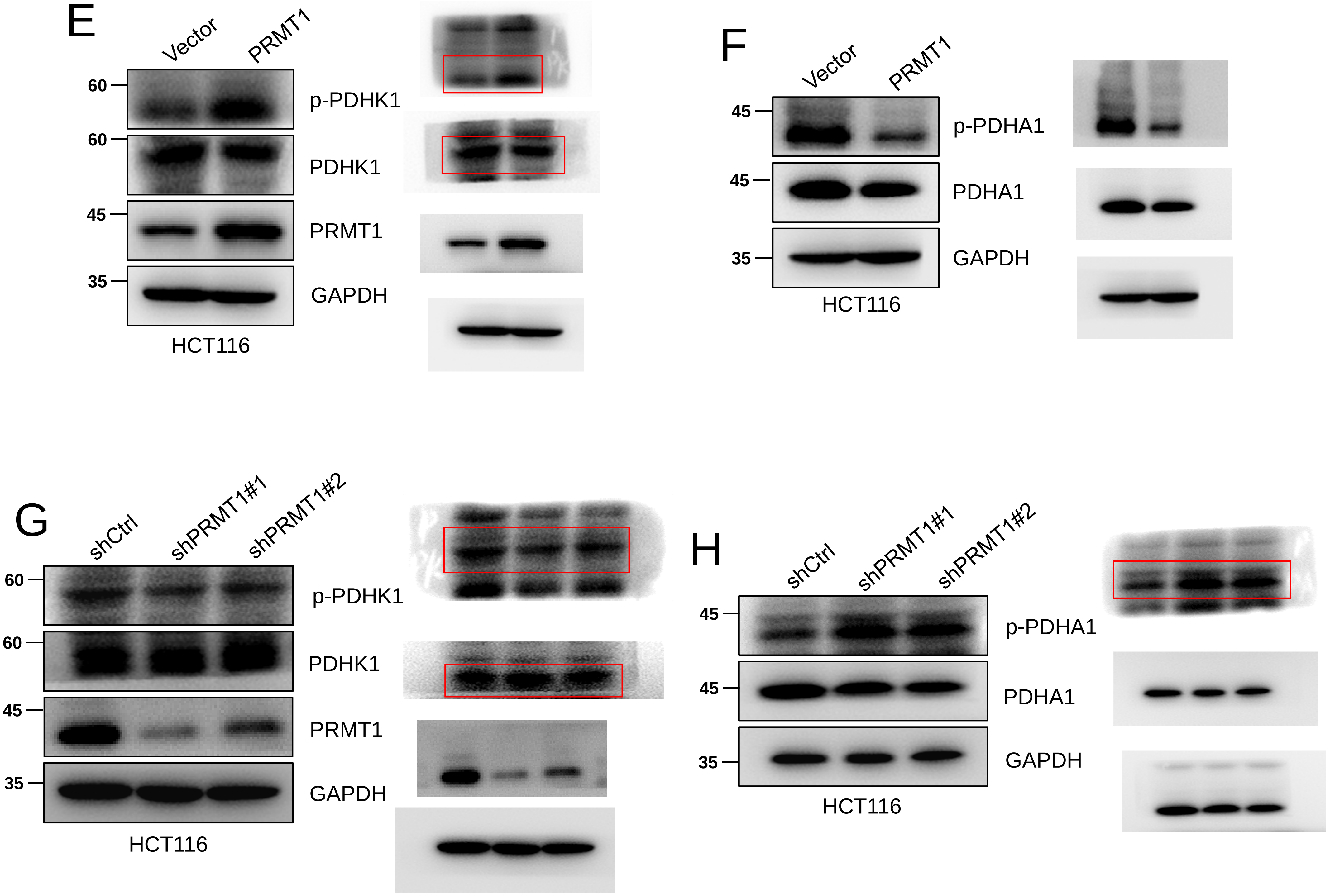
E
Vector
PRMT1
60
p-PDHK1
60
PDHK1
45
PRMT1
35
GAPDH
HCT116
F
Vector
PRMT1
45
p-PDHA1
45
PDHA1
35
GAPDH
HCT116
G
shCtrl
shPRMT1#1
shPRMT1#2
60
p-PDHK1
60
PDHK1
45
PRMT1
35
GAPDH
HCT116
H
shCtrl
shPRMT1#1
shPRMT1#2
45
p-PDHA1
45
PDHA1
35
GAPDH
HCT116

Figure 4

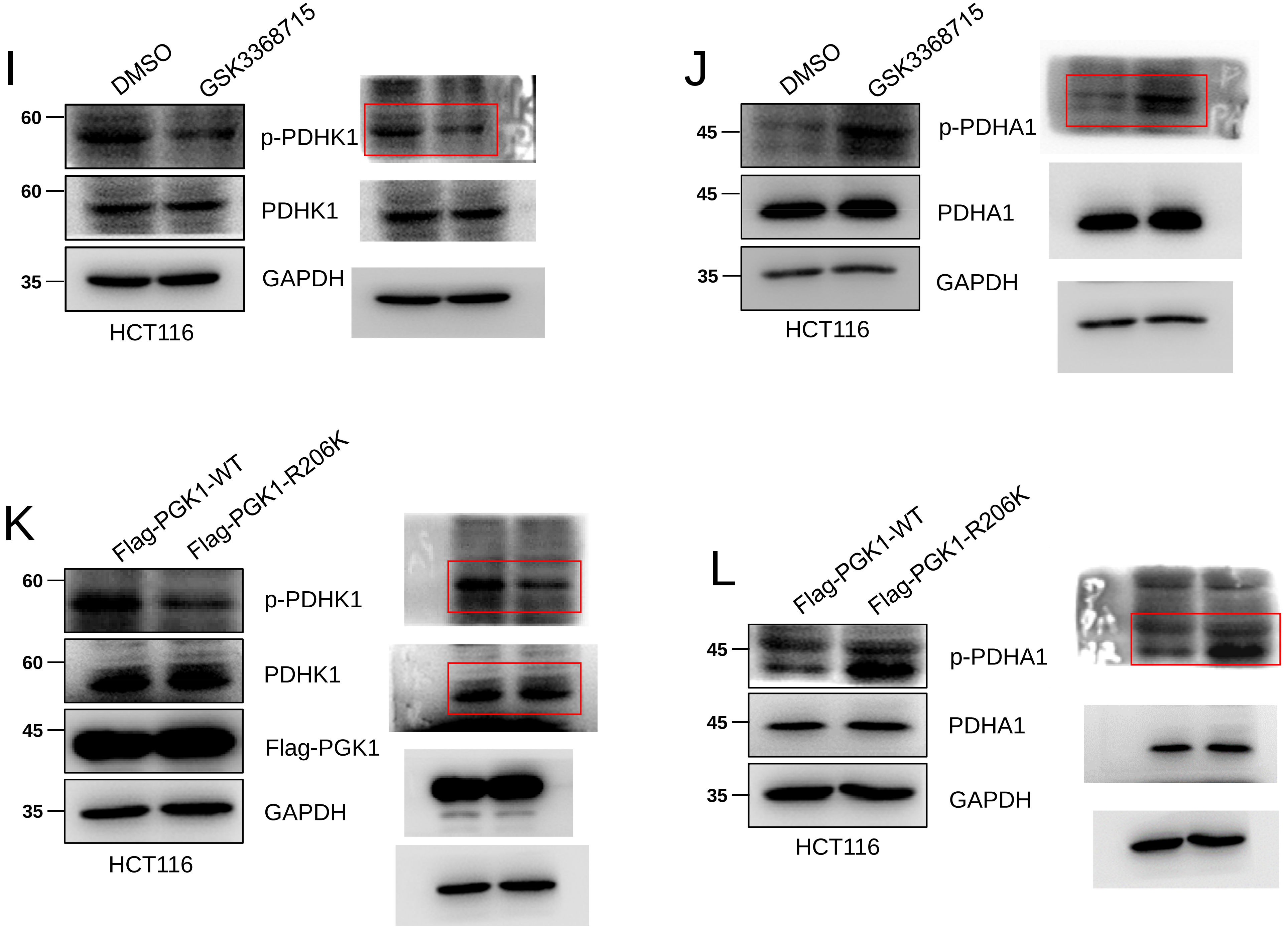

I
DMSO
GSK3368715
60
p-PDHK1
60
PDHK1
35
GAPDH
HCT116
J
DMSO
GSK3368715
45
p-PDHA1
45
PDHA1
35
GAPDH
HCT116
K
Flag-PGK1-WT
Flag-PGK1-R206K
60
p-PDHK1
60
PDHK1
45
Flag-PGK1
35
GAPDH
HCT116
L
Flag-PGK1-WT
Flag-PGK1-R206K
45
p-PDHA1
45
PDHA1
35
GAPDH
HCT116

Figure 4

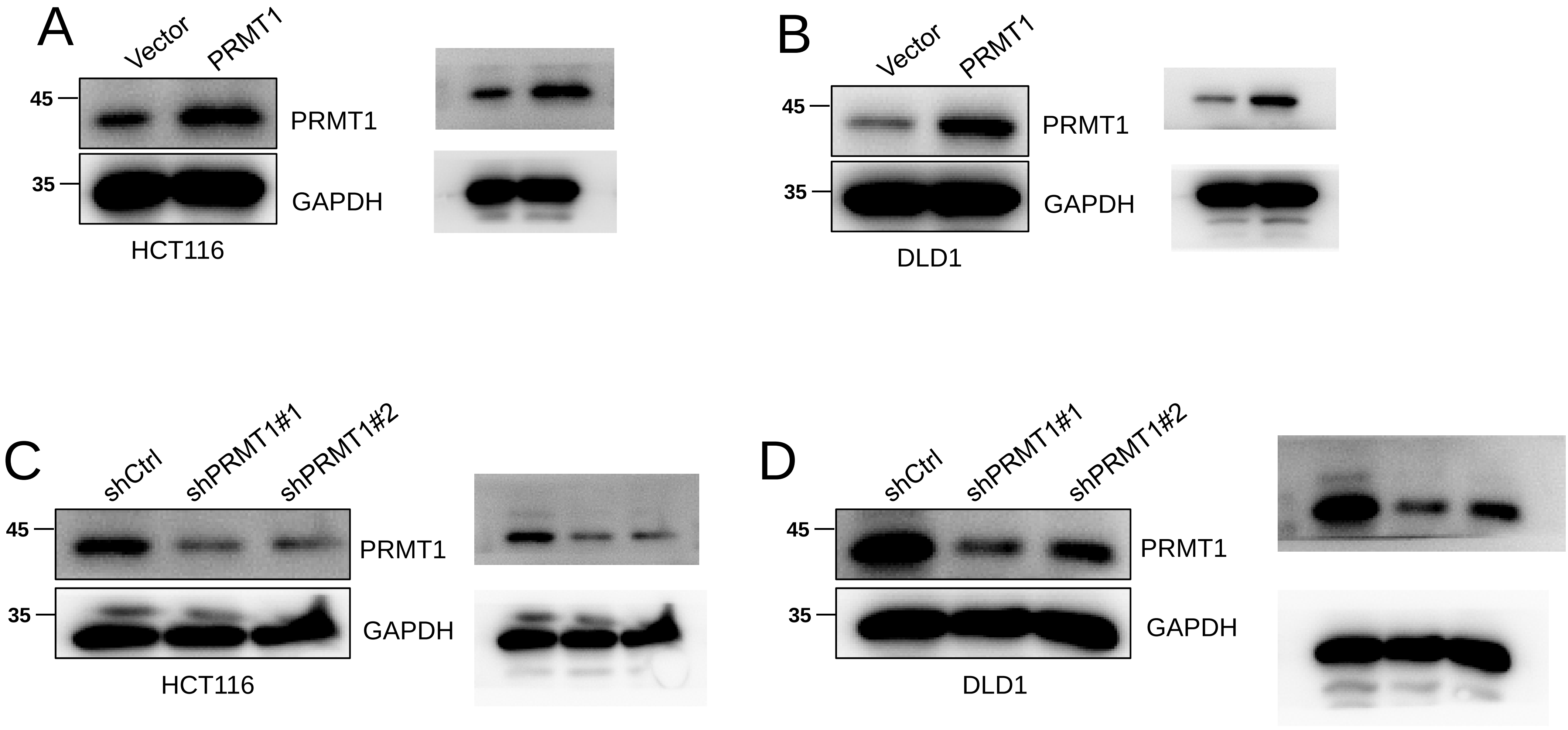

Supplementary Figure 1

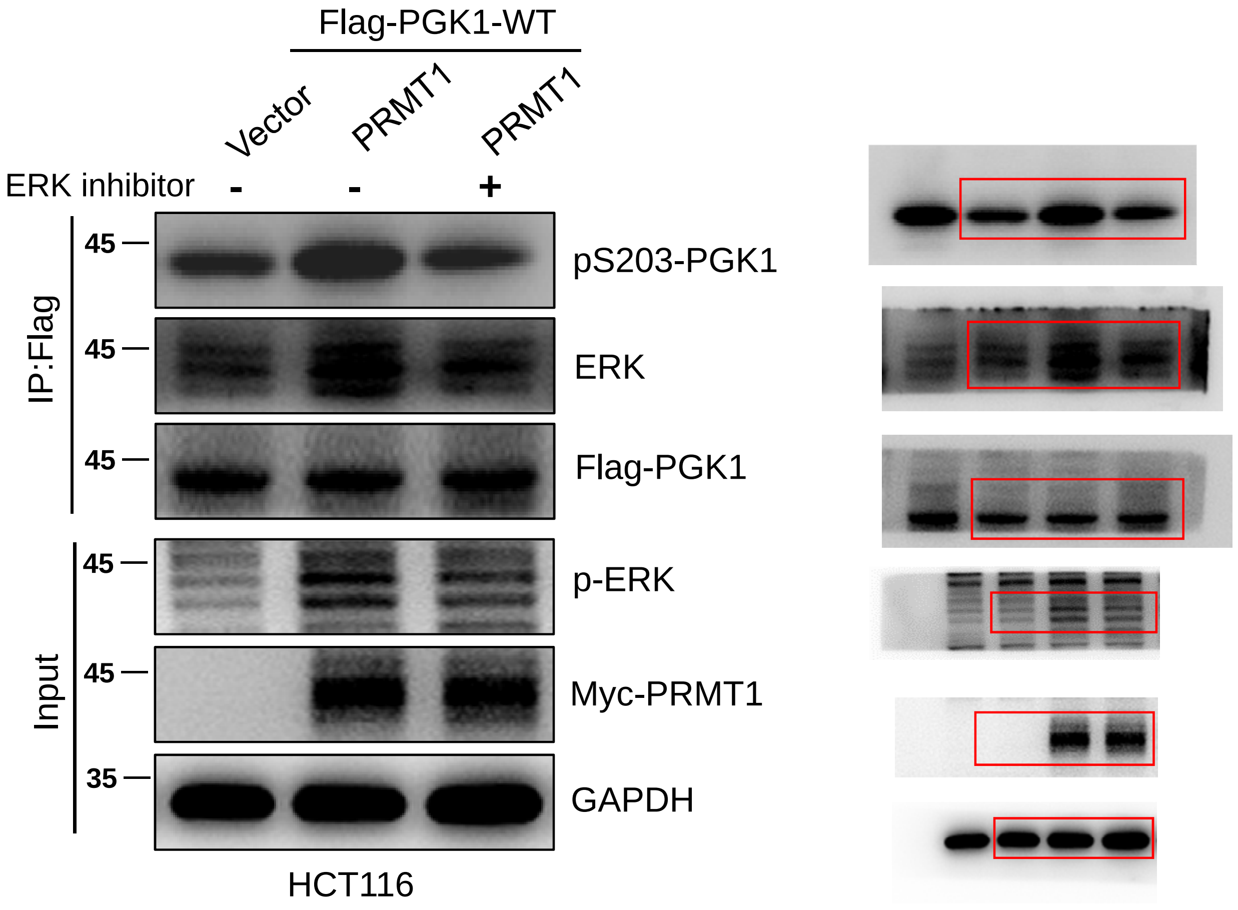

Supplementary Figure 2

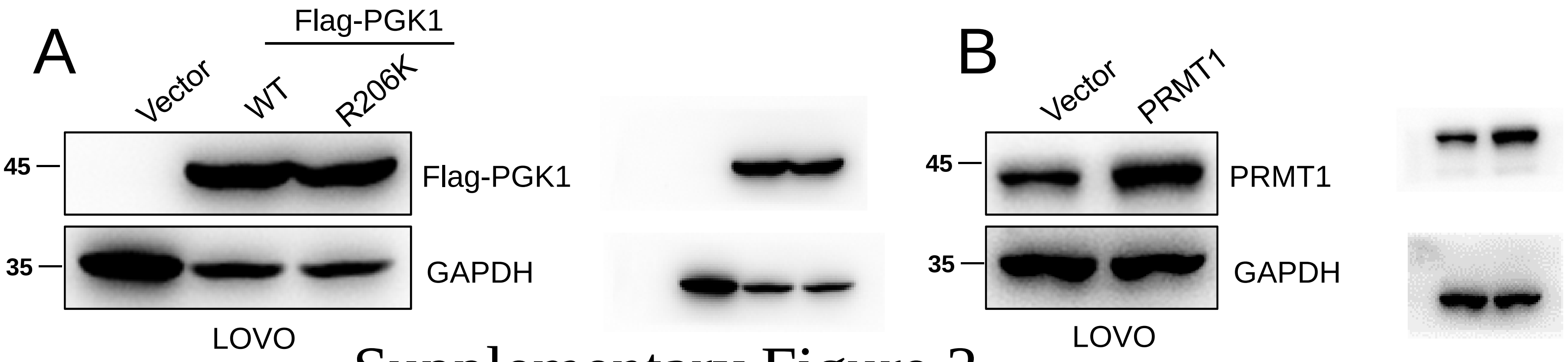

Supplementary Figure 3
